# Deciphering the Immunomodulatory Function of GSN ^+^ Inflammatory Cancer‐Associated Fibroblasts in Renal Cell Carcinoma Immunotherapy: Insights From Pan‐Cancer Single‐Cell Landscape and Spatial Transcriptomics Analysis

**DOI:** 10.1111/cpr.70062

**Published:** 2025-05-15

**Authors:** Shan Li, Xinwei Zhou, Haoqian Feng, Kangbo Huang, Minyu Chen, Mingjie Lin, Hansen Lin, Zebing Deng, Yuhang Chen, Wuyuan Liao, Zhengkun Zhang, Jinwei Chen, Bohong Guan, Tian Su, Zihao Feng, Guannan Shu, Anze Yu, Yihui Pan, Liangmin Fu

**Affiliations:** ^1^ Department of Urology The Second Xiangya Hospital of Central South University Changsha Hunan China; ^2^ Uro‐Oncology Institute of Central South University Changsha Hunan China; ^3^ Department of Urology Children's Hospital of Chongqing Medical University Chongqing China; ^4^ Department of Urology The First Affiliated Hospital of Sun Yat‐Sen University Guangzhou Guangdong China; ^5^ Department of Urology Sun Yat‐Sen University Cancer Center Guangzhou China; ^6^ State Key Laboratory of Oncology in South China, Guangdong Provincial Clinical Research Center for Cancer Sun Yat‐Sen University Cancer Center Guangzhou China; ^7^ Department of Geniturinary Oncology Tianjin Medical University Cancer Institute and Hospital, National Clinical Research Center for Cancer, Key Laboratory of Cancer Prevention and Therapy, Tianjin's Clinical Research Center for Cancer Tianjin China; ^8^ Department of Pediatric Intensive Care Unit (PICU) Guangdong Provincial People's Hospital Heyuan Hospital Heyuan Guangdong China; ^9^ Department of Urology, Guangzhou Women and Children's Medical Center Guangzhou Medical University, Guangzhou Institute of Pediatrics, Guangdong Provincial Clinical Research Center for Child Health Guangzhou China; ^10^ Department of Urology The Third Affiliated Hospital of Soochow University Changzhou China; ^11^ Key Laboratory of Diabetes Immunology (Central South University), Ministry of Education National Clinical Research Center for Metabolic Disease Changsha Hunan China

**Keywords:** cancer‐associated fibroblasts, clear cell renal cell carcinoma, immune checkpoint inhibitors, machine learning, multi‐omics analysis, pan‐cancer analysis

## Abstract

The heterogeneity of cancer‐associated fibroblasts (CAFs) could affect the response to immune checkpoint inhibitor (ICI) therapy. However, limited studies have investigated the role of inflammatory CAFs (iCAFs) in ICI therapy using pan‐cancer single‐cell RNA sequencing (scRNA‐seq) and spatial transcriptomics sequencing (ST‐seq) analysis. We performed pan‐cancer scRNA‐seq and ST‐seq analyses to identify the subtype of GSN^+^ iCAFs, exploring its spatial distribution characteristics in the context of ICI therapy. The pan‐cancer scRNA‐seq and bulk RNA‐seq data are incorporated to develop the Caf.Sig model, which predicts ICI response based on CAF gene signatures and machine learning approaches. Comprehensive scRNA‐seq analysis, along with in vivo and in vitro experiments, investigates the mechanisms by which GSN^+^ iCAFs influence ICI efficacy. The Caf.Sig model demonstrates well performances in predicting ICI therapy response in pan‐cancer patients. A higher proportion of GSN^+^ iCAFs is observed in ICI non‐responders compared to responders in the pan‐cancer landscape and clear cell renal cell carcinoma (ccRCC). Using real‐world immunotherapy data, the Caf.Sig model accurately predicts ICI response in pan‐cancer, potentially linked to interactions between GSN^+^ iCAFs and CD8^+^ Tex cells. ST‐seq analysis confirms that interactions and cellular distances between GSN^+^ iCAFs and CD8^+^ exhausted T (Tex) cells impact ICI efficacy. In a co‐culture system of primary CAFs, primary tumour cells and CD8^+^ T cells, downregulation of GSN on CAFs drives CD8^+^ T cells towards a dysfunctional state in ccRCC. In a subcutaneously tumour‐grafted mouse model, combining GSN overexpression with ICI treatment achieves optimal efficacy in ccRCC. Our study provides the Caf.Sig model as an outperforming approach for patient selection of ICI therapy, and advances our understanding of CAF biology and suggests potential therapeutic strategies for upregulating GSN in CAFs in cancer immunotherapy.

## Graphical abstract

Pan‐cancer scRNA‐seq and ST‐seq analyses identify GSN^+^ inflammatory cancer‐associated fibroblasts (CAFs) and their spatial distribution in immune checkpoint inhibitor (ICI) therapy. The Caf.Sig model accurately predicts ICI response across cancers using CAF gene signatures and machine learning. Higher GSN^+^ inflammatory CAF proportions correlate with ICI non‐response in pan‐cancer and clear cell renal cell carcinoma (ccRCC). Spatial transcriptome analysis confirms interactions between GSN^+^ inflammatory CAFs and CD8^+^ Tex cells impact ICI efficacy. GSN downregulation in CAFs drives CD8^+^ T cells towards a dysfunctional state in ccRCC.

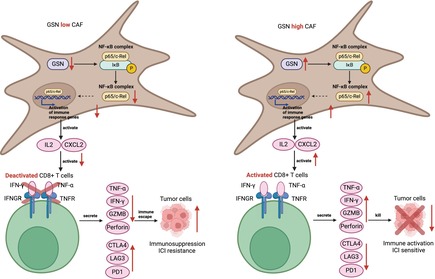

## 1 Introduction

Immune checkpoint inhibitor (ICI) therapies are increasingly central to cancer treatment strategies, with proven efficacy against various tumours, involving lung cancer [1], melanoma [2] and gastrointestinal cancer [3]. Nevertheless, alongside therapeutic side effects, the low response rate remains a huge challenge limiting the broader application of immunotherapy [4]. This has driven biomarker research to predict immunotherapy responses and optimise combination treatments to overcome therapy resistance. Previous biomarker research often relies on whole‐exome sequencing or RNA sequencing (RNA‐seq) of various cancer samples, which solely capture the overall genetic profile of cancers [5], such as programmed cell death‐ligand 1 (PD‐L1) expression [6], microsatellite instability (MSI) [7] and tumour mutation burden (TMB) [6]. The emergence of single‐cell RNA‐seq (scRNA‐seq) now allows mRNA expression analysis in single cell resolution, enabling the discovery of more predictive biomarkers in the future.

Among the varied cell subpopulations in the tumour microenvironment (TME), cancer‐associated fibroblasts (CAFs) have emerged as a dominant and numerous group [8], attracting considerable scientific interest recently. In the TME, CD4^+^ T cells and CD8^+^ T cells predominantly act as effector cells in ICI therapy, with their degree of infiltration being used as a reliable predictor for response to ICIs [9]. However, the majority of patients have an immune‐exclusion phenotype, and according to recent research, CAFs play a key mediator function in this phenomenon [10]. The nuanced interplay between CAFs, stromal elements and immune components critically shapes the restructuring of TME during immunotherapy. This procedure involves the formation of emerging blood vessels, extracellular matrix (ECM) remodelling, promotion of epithelial‐mesenchymal transition and mechanisms for escaping immune surveillance [11]. Previous studies indicate that matrix CAFs could create a compact ECM network at the tumour‐stroma interface contributing to T cell exclusion, and propose that immunomodulatory CAFs are crucial for regulating immune cell infiltration and immune detection [12]. Importantly, current therapeutic strategies, encompassing both immune‐targeted therapies and cytotoxic drugs, often overlook the significant role of CAFs. Recent research has shown that interactions between CAFs and malignant cells contribute to chemotherapy resistance and immunotherapy response across multiple cancer types [13, 14, 15, 16]. However, the specific mechanisms by which CAF subgroups are correlated to ICI treatment resistance in clear cell renal cell carcinoma (ccRCC) remain unclear.

The medical study landscape is undergoing a transformative change, driven by state‐of‐the‐art bioinformatics approaches. Innovative techniques in gene expression analysis, genetic variation mapping and high‐resolution scRNA‐seq and spatial transcriptomics sequencing (ST‐seq) are revolutionising our comprehension of biological mechanisms. These methods, especially when utilised in the research of GSN^+^ inflammatory CAFs (iCAFs) in ICI therapy, provide novel insights into potential therapeutic strategies. Utilising multiple machine learning (ML) algorithms [17], we integrated high‐quality scRNA‐seq and bulk RNA‐seq datasets to construct the Caf.Sig model, which showed predictive potential in pan‐cancer responsiveness to immunotherapy. In ccRCC, the interactions between GSN^+^ iCAFs and CD8^+^ exhausted T (Tex) cells were identified as a potential mechanism influencing immunotherapy efficacy, confirmed by in vitro and in vivo experiments.

## 2 Materials and Methods

### 2.1 Pan‐Cancer scRNA‐Seq Data Acquisition and Analysis

Nine immunotherapy cohorts, comprising both ICI response information and scRNA‐seq data, were analysed to explore the association of CAFs and ICI effectiveness. Those datasets were sourced from publicly available repositories, including skin cutaneous melanoma (SKCM, GSE120575 [18] and GSE115978 [19]), renal cell carcinoma (RCC, SCP1288 [20]), urothelial carcinoma (UC, GSE145281 [21]), triple negative breast cancer (TNBC, GSE169246 [22]), breast invasive carcinoma (BRCA, Bassez 2021 [23]), carcinoma of colon and rectum (CRC, GSE205506 [24]), non‐small cell lung carcinoma (NSCLC, GSE207422 [25]) and basal cell carcinoma (BCC, GSE123813 [26]). Patients exhibiting partial response or complete response were categorised as responders, while those with progressive disease or stable disease were defined as non‐responders. The scRNA‐seq analysis was performed with ‘Seurat’ R package [27], which facilitated cell annotation and the extraction of CAFs specific data from each cohort. We deleted low‐quality cells based on the criteria of > 40,000 UMI per cell, < 500 genes per cell, > 5000 genes per cell and > 20% mitochondrial genes. Doublets were eliminated by ‘DoubletFinder’ R package [28]. Raw counts were normalised using NormalizeData, and 2000 highly variable genes were identified with FindVariableFeatures. The ‘Harmony’ R package was employed to integrate the datasets and remove batch effects [29]. Within the maker genes of CAF subpopulations [30, 31], various clusters of CAFs were identified. Differentially expressed genes between responders and non‐responders were identified using the ‘FindMarkers’ function, with thresholds set at log_2_ fold change (log_2_FC) > 0 and an adjusted *p*‐value < 0.05. The ‘Monocle’ R package was utilised to construct cell developmental trajectories, visualising the distribution of responders and non‐responders within the trajectory space [32]. Seven single cell scoring algorithms (AUCell in ‘AUCell’ R package, Ucell in ‘Ucell’ R package, Single‐sample Gene Set Enrichment Analysis [ssGSEA] and GSVA in ‘GSVA’ R package, singscore in ‘singscore’ R package, AddModuleScore and PercentageFeatureSet in ‘Seurat’ R package) were employed to conduct enrichment scoring [27, 33, 34, 35]. Functional enrichment analysis based on Gene Ontology (GO), Kyoto Encyclopaedia of Genes and Genomes (KEGG) and WikiPathway terms in scRNA‐seq data were performed via ‘SCP’ R package (https://github.com/zhanghao‐njmu/SCP). ‘CellChat’ R package was used to analyse the cellular communication network among cell subpopulations [36]. The ‘pySCENIC’ (version 0.11.2) algorithm with Python (version 3.7) was used to investigate enriched transcription factors (TF) and regulon activities of cell subpopulations, building TF regulatory networks and identifying stable cell states [37].

### 2.2 Source Data of Pan‐Cancer RNA‐Seq Cohorts

Genomic data, clinical information and dysfunction score for kidney renal clear cell carcinoma (KIRC) from The Cancer Genome Atlas (TCGA) were downloaded from UCSC Xena. To develop a robust Caf.Sig model, we comprehensively obtained RNA‐seq data and clinical characteristics of immunotherapy‐treated patients from 14 ICI cohorts, involving five SKCM cohorts (Hugo 2016 [38], Liu 2019 [39], Gide 2019 [40], Riaz 2017 [41] and Van Allen 2015 [42]), four RCC cohorts (Braun 2020 [43], Ascierto 2016 [44], Powles 2017 [45] and Rini 2019 [46]), two UC cohorts (Mariathasan 2018 [47], Synder 2017 [48]), one glioblastoma multiform (GBM) cohort (Zhao 2019 [49]), one gastric cancer (GC) cohort (Kim 2018 [50]) and one NSCLC cohort (Jung 2019 [51]). Sequencing data were log_2_‐transformed, and batch effects between cohorts were corrected using the ‘sva’ R package [52]. We used Braun 2020, Liu 2019, Gide 2019, Riaz 2017 and Mariathasan 2018 as the training cohort to develop Caf.Sig to predict not responding to immunotherapy. Hugo 2016, Van Allen 2015, Synder 2017, Kim 2018 and Zhao 2019 served as the validation cohort, while Ascierto 2016, Powles 2017, Rini 2019 and Jung 2019 were incorporated as the external validation cohort.

### 2.3 Spatial Transcriptomics Analysis

To investigate the biological functions of CAFs in ICI in the ST‐seq context, we downloaded ST‐seq data of hepatocellular carcinoma (HCC) patients being treated with anti‐PD‐1 therapy [53]. To precisely determine the cellular makeup at each location on the 10x ST‐seq slide, deconvolution analysis was utilised [54]. This method, grounded in both ST and scRNA‐seq data, specifically accounted for the relevant tumour type. In parallel, to pinpoint the spatial coordinates of CAFs, we conducted an integrated analysis of scRNA‐seq and ST‐seq data using the ‘CellTrek’ R package [55] with its standard settings. The spatial k‐distance between distinct CAF subtypes and cell subpopulations was computed using the run_kdist function. We computed the spatial k‐distance among various cell subpopulations within every tissue section, ranked them from nearest to farthest, and then applied the robust rank aggregation (RRA) algorithm to generate an overall ranking of every cell type [56]. ‘SPATA2’ R package was utilised to investigate the dynamic biological processes and the spatial trajectories across high‐density regions of various cell subpopulations at the spatial resolution [57]. ‘SpaTalk’ R package was used to identify intercellular communication patterns within the spatial environment [58].

### 2.4 Establishment of Caf.Sig Model and Caf.Sig Score

Utilising marker genes of CAFs with predictive value of ICI efficacy, we established Caf.Sig Model to predict responsiveness to ICI in pan‐cancer bulk‐seq data. We incorporated 12 ML algorithms including random forest (RF), Lasso, Ridge, elastic net (Enet), stepwise Glm, GlmBoost, linear discriminant analysis (LDA), partial least squares regression for Glm (plsRglm), generalised boosted regression modelling (GBM), extreme gradient boosting (XGB), support vector machine (SVM) and Naive Bayes for the prediction of ICI effectiveness. Then totally 113 predictive ML algorithm combinations were trained in the training cohort, based on leave‐one‐out cross‐validation (LOOCV) framework to construct Caf.Sig Model. Models with less than five model genes were omitted. The average area under the curve (AUC) was computed in every ML combination in the training, internal test and external test sets [17]. The predictive ML combination with the top average AUC was elected as the best gene signature. The Caf.Sig risk scores were calculated by a linear combination approach via incorporating gene expression profiles from various feature selection methods. To further appraise the performances of Caf.Sig model, 13 ICI‐related signatures were involved (Stem.Sig [59], IMS.Sig [60], CRMA.Sig [61], ImmmunCells.Sig [62], IFNG.Sig [63], T.cell.infamed.Sig [63], PDL1.Sig [64], TcellExc.Sig [19], NLRP3.Sig [65], Cytotoxic.Sig [66], TRS.Sig [67], LRRC15.CAF.Sig [68], IMPRES.Sig [69] and IPRES.Sig [38]) to compare their performances. The codes and algorithms associated with those signatures were obtained from the initial research. We assessed the expression levels of every gene in the Caf.Sig model within multiple ICI bulk‐seq datasets based on immunotherapy response. Utilising ssGSEA algorithm, we identified upregulated and downregulated genes in non‐responders and computed the Caf.Sig score based on the formula: Caf.Sig score = ssGSEA_Score (up‐regulated) − ssGSEA_Score (down‐regulated). The Caf.Sig score calculation by AUCell, Ucell, GSVA, singscore, AddModuleScore and PercentageFeatureSet algorithms was performed by: Caf.Sig score = Algorithm_Score (up‐regulated) − Algorithm_Score (down‐regulated).

### 2.5 Model Verification in Precision, Stability and Reliability

To thoroughly assess the accuracy, stability and reproducibility of the Caf.Sig Model, a series of comprehensive validation methods were employed. The model's accuracy was evaluated using a confusion matrix via the ‘cvms’ R package. Additionally, Receiver Operating Characteristic (ROC) curves, calibration curves and decision curve analysis (DCA) were implemented to gauge the model's precision, discrimination ability and clinical utility. The model's predictive capacity was then compared with traditional clinical features through ROC analysis. Furthermore, univariate and multivariate logistic regression analyses were conducted to affirm the model's independent predictive value. Finally, a nomogram was constructed to visually represent the predictive strength of the Caf.Sig Model.

### 2.6 Function Enrichment Analysis

Differentially expressed genes (DEGs) were defined within two groups divided by the median gene expression. DEGs were calculated with the ‘limma’ R package, via a false discovery rate threshold of < 0.05 and an absolute log_2_ fold change (FC) of > 1. The functional enrichment of DEGs was conducted in GO and KEGG databases by the ‘clusterprofiler’ R package [70]. The ssGSEA analysis on every tumour sample was conducted by ‘GSVA’ R package [35], within the ‘h.all.v7.4.symbols.gmt’ gene set from MSigDB. Gene set enrichment analysis (GSEA) was applied to explore the molecular pathways associated with different groups [71], with a threshold of *p* < 0.05 and Normalised Enrichment Score > 1.

### 2.7 Immunohistochemistry Staining

To verify the different expressions of GSN associated with ICI efficacy, we performed immunohistochemistry (IHC) staining in tissue samples of three non‐responders and three responders from the Department of Urology, the First Affiliated Hospital, Sun Yat‐sen University (Guangzhou, China). ccRCC samples were paraffin‐embedded and sectioned into 4 mm slices. Following dewaxing, hydration and antigen retrieval, the tissues were incubated overnight at 4°C with primary antibody: Gelsolin Polyclonal antibody (11644‐2‐AP, Protein tech). Subsequent steps included incubation with a Goat anti‐Rabbit IgG secondary antibody (ZENBIO, China), DAB staining (ZENBIO, China) and blocking. Staining figures were captured with a microscope. Every sample was assessed for staining intensity (0: none, 1: mild, 2: moderate, 3: strong) and the percentage of positive cells (0: 0%, 1: 1%–25%, 2: 26%–50%, 3: 51%–75%, 4: 76%–100%). The final IHC scores were the sum of intensity and percentage scores.

### 2.8 Isolation of Primary RCC Cells and CAFs

Tumour tissues from HLA‐A2^+^ ccRCC patients with pathological diagnosis were isolated and washed in cold PBS (supplemented with 2% penicillin–streptomycin solution). The tissues were then cut into pieces and digested in DMEM containing 0.002% DNase I (Stemcell, Canada), 0.01% hyaluronidase (Stemcell, Canada), 0.2% collagenase IV (Stemcell, Canada) and 3 × 10–3 m CaCl 2 (21115, Sigma‐Aldrich) at 37°C for 60 min with constant shaking at 200 rpm. The digestion was centrifuged at 300*g*, and the supernatant was filtered through a 40 μm cell strainer (352340, Corning). The cell suspension was cultured in a six‐well plate with DMEM containing 10% FBS and 1% penicillin–streptomycin solution.

### 2.9 Primary Human Peripheral Blood Mononuclear Cell and CD8 ^+^ T Cell Isolation

Peripheral blood mononuclear cells (PBMCs) were isolated from the same HLA‐A2^+^ ccRCC patients by FicollPaque (17‐5442‐02, GE Healthcare) density gradient centrifugation under the manufacturer instructions, and PBMCs were washed twice by PBS. Primary human CD8^+^ T cells were purified with a CD8^+^ T cell Isolation Kit (Miltenyi, 130‐096‐495) under the manufacturer instructions. CD8^+^ T cells were verified to be > 95% pure via flow cytometry analysis.

### 2.10 Co‐Culture Assay

Primary RCC cells and isolated CD8^+^ T cells were cocultured with siCtrl or siGSN‐transfected primary CAF cells in Gibco DMEM. In the co‐cultured system, western blotting was conducted for protein detection and flow cytometry was employed to investigate the functions of CD8^+^ T cells. Human CD8^+^ T cells were purified with a Human CD8^+^ T cell isolation kit (Catalogue # 100‐0202, Stemcell, Canada), and the selection criteria was that the final product was > 95% CD8^+^ T cells. For flow cytometry, the cells were collected, washed and blocked by PBS containing 1% BSA solution. After incubation with the related antibodies, the cells were further analysed.

### 2.11 Quantitative Real‐Time PCR Analyses

Total RNA from primary CAFs from tumour tissue was extracted by TRIzol (Invitrogen). Total RNA was reverse transcribed with 4 × Reverse Transcription Master Mix (EZBioscience, USA) under the manufacturer's instruction. Quantitative real‐time PCR was performed using 2 × SYBR Green qPCR Master Mix (EZBioscience, USA) and a Roche LightCycler 480 Instrument. The forward and reverse primers used for GAPDH are TGCACCACCAACTGCTTAGC and GGCATGGACTGTGGTCATGAG. The forward and reverse primers used for GSN are GGTGTGGCATCAGGATTCAAG and TTTCATACCGATTGCTGTTGGA. Two GSN siRNAs (si‐1: UGAAGAAGUCUCCAUAAAGGU CUUUAUGGAGACUUCUUCACG; si‐2: AAGAUUUUCCCAUCUUUGCCG GCAAAGAUGGGAAAAUCUUUG) were synthesised by RiboBio (Beijing, China).

### 2.12 Western Blotting Analysis

Cells were lysed in cell lysis buffer (Beyotime, China) with protease inhibitor cocktail (CoWin Biosciences, China) on ice and then collected by cell scrapers (BIOFIL, China). Protein quantitation was conducted by Pierce BCA Protein Assay Kit (ThermoFisher, USA) and measured at a wavelength of 562 nm (MD VersaMax, USA). Protein samples were loaded in 7.5%–12.5% SDS–PAGE gels. After electrophoresis, proteins were transferred to PVDF membranes (Merck Millipore, USA) in an electrophoretic transfer unit (Tanon, China). After blocking, the PVDF membranes were incubated with primary antibodies at 4°C for more than 12 h. After 1 h of incubation with secondary antibodies at room temperature, the protein bands were detected by chemiluminescence (Tanon). These primary antibodies were used in our western blot analysis: p65 (8242, Cell Signalling Technology, CST), p‐p65 (3033, CST), IKB (4812, CST), p‐IKB (2859, CST), IKKα + β (2697, CST), anti‐human GSN (11644‐2‐AP, Protein tech), β‐tubulin (10094‐1‐AP, Protein tech).

### 2.13 Flow Cytometry

In the co‐culture experiment, T cells from the co‐culture of mouse tumour cells with immune cells, as well as T cells present in the culture medium, were collected. Mouse tumour tissues were processed using enzymatic digestion to obtain a single‐cell suspension with tumour‐associated T cells. After filtering, T cells were resuspended in PBS containing 5 × 10–3 m EDTA and 1% FBS. For detection of cytotoxic cytokine production, cells were treated with 50 ng mL‐1 phorbol 12‐myristate 13‐acetate, 1 × 10^−6^ m ionomycin and protein transport inhibitor (BD) for 6 h at 37°C. Cells were then fixed and permeabilized with a Fixation and Permeabilization Solution Kit (BD, 554714) following the manufacturer's instructions, and cells were then stained with the indicated primary antibodies. Cell surface Fc receptors were blocked using TruStain FcX (Catalogue No. 101319, BioLegend) to prevent non‐specific binding of antibodies to Fc receptors. This was followed by stimulation inhibition using the Cell Activation Cocktail (with Brefeldin A, Catalogue No. 423303, BioLegend). Samples were analysed with a Beckman CytoFLEX Flow cytometer (Beckman Coulter, USA), and FlowJo10 software was used to analyse the data. The following antibodies were used for flow cytometry analyses: AF700 anti‐human CD45 (304024, BioLegend), APC‐Cy7 anti‐mouse CD45 (557659, BioLegend), APC anti‐human CD3 (317317, BioLegend), PE‐Cy7 anti‐mouse CD3 (552774, BioLegend), FITC anti‐human CD8a (300905, BioLegend), PE anti‐mouse CD8a (162303, BioLegend), FITC anti‐mouse CD8a (100705, BioLegend), PE‐Cy7 anti‐human TNF‐α (502930, BioLegend), PerCP/Cy5.5 anti‐human Perforin (308113, BioLegend), PE anti‐mouse Perforin (154405, BioLegend), AF647 anti‐human/mouse Granzyme B (515406, BioLegend), PE anti‐human/mouse Granzyme B (372207, BioLegend), PE anti‐human IFN‐γ (383304, BioLegend), BV650 anti‐mouse IFN‐γ (505831, BioLegend), PerCP/Cy5.5 anti‐mouse PD‐1 (109120, BioLegend), BV605 anti‐mouse LAG‐3 (125257, BioLegend), BV421 anti‐mouse CTLA‐4 (106312, BioLegend), BV510 Zombie Aqua Fixable Viability Kit (423101, BioLegend).

### 2.14 Cytotoxicity Assays

CD8^+^ T cells were generated as described above and cocultured with primary kidney tumour cells and CAFs at an effector/target (E/T) ratio of 10:1 in 48‐well plates for 12 h at 37°C. Tumour cells were then stained with PI (ST511, Beyotime) and immediately analysed via flow cytometry. T cells were collected and then treated with protein transport inhibitor (BD) for 6 h at 37°C, followed by fixation and permeabilization with a Fixation and Permeabilization Solution Kit (554714, BD) under the manufacturer's instruction. Cells were stained with PE anti‐human IFN‐γ (383304, BioLegend), AF647 anti‐human/mouse Granzyme B (515406, BioLegend), PE‐Cy7 anti‐human TNF‐α (502930, BioLegend) and PerCP/Cy5.5 anti‐human Perforin (308113, BioLegend). Samples were examined by a Beckman CytoFLEX Flow cytometer (Beckman Coulter, USA), and FlowJo10 software was utilised to investigate the data.

### 2.15 In Vivo Mouse Experiments

The in vivo mouse experiments were approved by the Institutional Animal Care and Use Committee of Sun Yat‐sen University and conducted in accordance with the guidelines for the care and use of animals. Six‐ to eight‐week‐old male BALB/c mice were purchased from GemPharmatech (China) and fed in standard pathogen‐free conditions. BALB/c mice were subcutaneously injected with Renca cells co‐cultured with 3T3 cells (5 × 10^5^ cells/100 μL). The GSN overexpression adeno‐associated virus (AAV) was established, verified and supplied by Geneseed Biotechnology (China). BALB/c mice were randomly divided into four groups: control group, AAV‐GSN group, PD‐1 treatment group and AAV‐GSN + PD‐1 treatment group. Mice were then administered GSN overexpression AAV via intratumor injection (5 nmol once a day for 7 days). For anti‐PD‐1 therapy, mice were intraperitoneally injected with anti‐PD‐1 antibody (BioXcell, USA) (100 μg per mouse twice a week). The palpable tumour weight was measured every week. The tumour volume (mm^3^) was computed as follows: tumour volume = (length × width^2^)/2. The mice were sacrificed as the tumour size reached 1500 mm^3^ or ulceration occurred. The tumours were separated surgically for flow cytometry.

### 2.16 Enzyme‐Linked Immunosorbent Assay

Blood samples were collected by a serum separator tube from mice before euthanasia. Subsequently, samples were allowed to clot at room temperature for 1 h, followed by centrifugation for 10 min at 3000*g*. Serum was transferred to a new tube and stored at −80°C before analysis. For coculture assays, supernatant from siCtrl or siGSN primary ccRCC cells and CAFs co‐cultured with CD8^+^ T cells was retrieved and stored at −80°C. IFN‐γ and TNF‐α levels in the serum samples were detected by a human IFN‐γ and TNF‐α ELISA kit (Elikine, USA).

## 3 Results

### 3.1 Pan‐Cancer Single Cell Transcriptome Atlas of ICI Treatment

**FIGURE 1:**
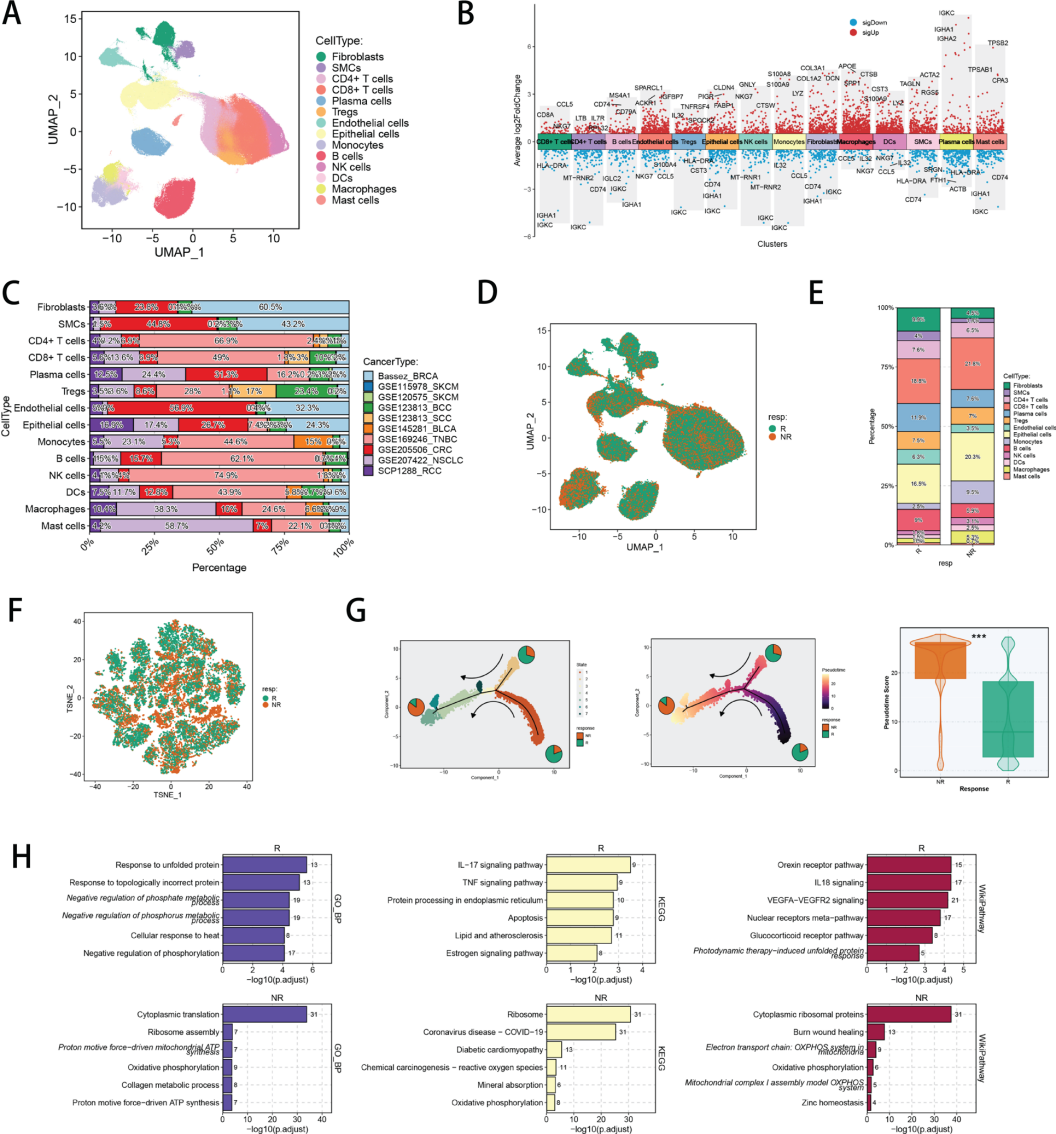
Major cell type landscapes of ICI related pan‐cancer scRNA‐seq cohorts. (A) Colour‐coded UMAP plot of major cell types in nine scRNA‐seq cohorts with ICI treatment. (B) Marker genes of each major cell type. (C) The cell proportion of every cohort in each major cell type. (D) The cell distribution of ICI responders and non‐responders in all cells. (E) The proportion of each major cell type between responders and non‐responders. (F) The cell distribution of ICI responders and non‐responders in fibroblsts. (G) Trajectory analysis of fibroblasts from ICI responders and non‐responders. (H) Biological Process of GO enrichment, KEGG analysis and WikiPathway enrichment analyses of DEGs between fibroblasts from ICI responders and non‐responders. DEGs, differential expressed genes; GO, Gene Ontology; ICI, immune checkpoint inhibitor; KEGG, Kyoto Encyclopaedia of Genes and Genomes; UMAP, uniform manifold approximation and projection. *p < 0.05; **p < 0.01; ***p < 0.001; ****p < 0.0001.

Nine scRNA‐seq datasets with ICI response information were utilised to study the association between CAFs and immunotherapy responsiveness. To identify various major cell types, we preformed uniform manifold approximation and projection (UMAP) to reduce the dimensions and cluster the single cells in nine scRNA‐seq cohorts including nine cancer types (Figure 1A). In terms of ICI responding, the total cell counts in responders and non‐responders sequenced by scRNA‐seq, respectively, varied remarkably across every cancer cohort (Figure S1A). Generally, 14 major cell types were distinguished in the integrated dataset, and the typical marker genes of every cell type proved the successful annotation of these cell populations (Figure 1B). Among all cell types, TNBC, BRCA, NSCLC and CRC comprised the largest proportion, and fibroblasts were shared by every cancer dataset (Figure 1C), indicating their universal presence in ICI treated tumour tissues. To understand the complexity in TME of responders and non‐responders, we utilised UMAP to visualise the different cells from responders and non‐responders (Figure 1D), as well as calculated the ratio of major cell types in two groups (Figure 1E). We discovered that the proportion of fibroblasts was significantly lower in responders, implying their potential mechanisms and functions which could affect ICI responsiveness in cancer patients. To deeply explore the effectiveness of fibroblasts on ICI efficacy, we next lowered the dimensions of fibroblasts by T‐distributed stochastic neighbour embedding (tSNE), which showed that fibroblasts from responders and non‐responders were distinctly divided, indicating their underlying effects on immunotherapy responsiveness (Figure 1F). Pseudotime analysis demonstrated that fibroblasts could differentiate into various subpopulations in the process of tumour progression, while fibroblasts from responders and non‐responders were clearly divided into two groups with significantly varied pseudotime scores, indicating that fibroblasts could differentiate into different trajectories among non‐responders and responders (Figure 1G, Figure S1B). To investigate the biological differences between responders and non‐responders, we analysed the gene expression profiles in fibroblasts and identified several genes, such as JUND, GPX7, UBB and NRN1, with significantly distinct expression levels (Figure S1C,D). Moreover, we performed Biological Process of GO, KEGG and WikiPathway function enrichment analysis with DEGs of fibroblasts. The upregulated genes in responders were enriched in ‘Response to unfolded protein’, ‘TNF signalling pathway’ and ‘IL18 signalling’, while the upregulated genes in non‐responders were enriched in ‘Cytoplasmic translation’, ‘Chemical carcinogenesis—reactive oxygen species’ and ‘Oxidative phosphorylation’ (Figure 1H).

### 3.2 Development and Validation of Caf.Sig Model to Predict ICI Response in Pan‐Cancer Landscape

**FIGURE 2:**
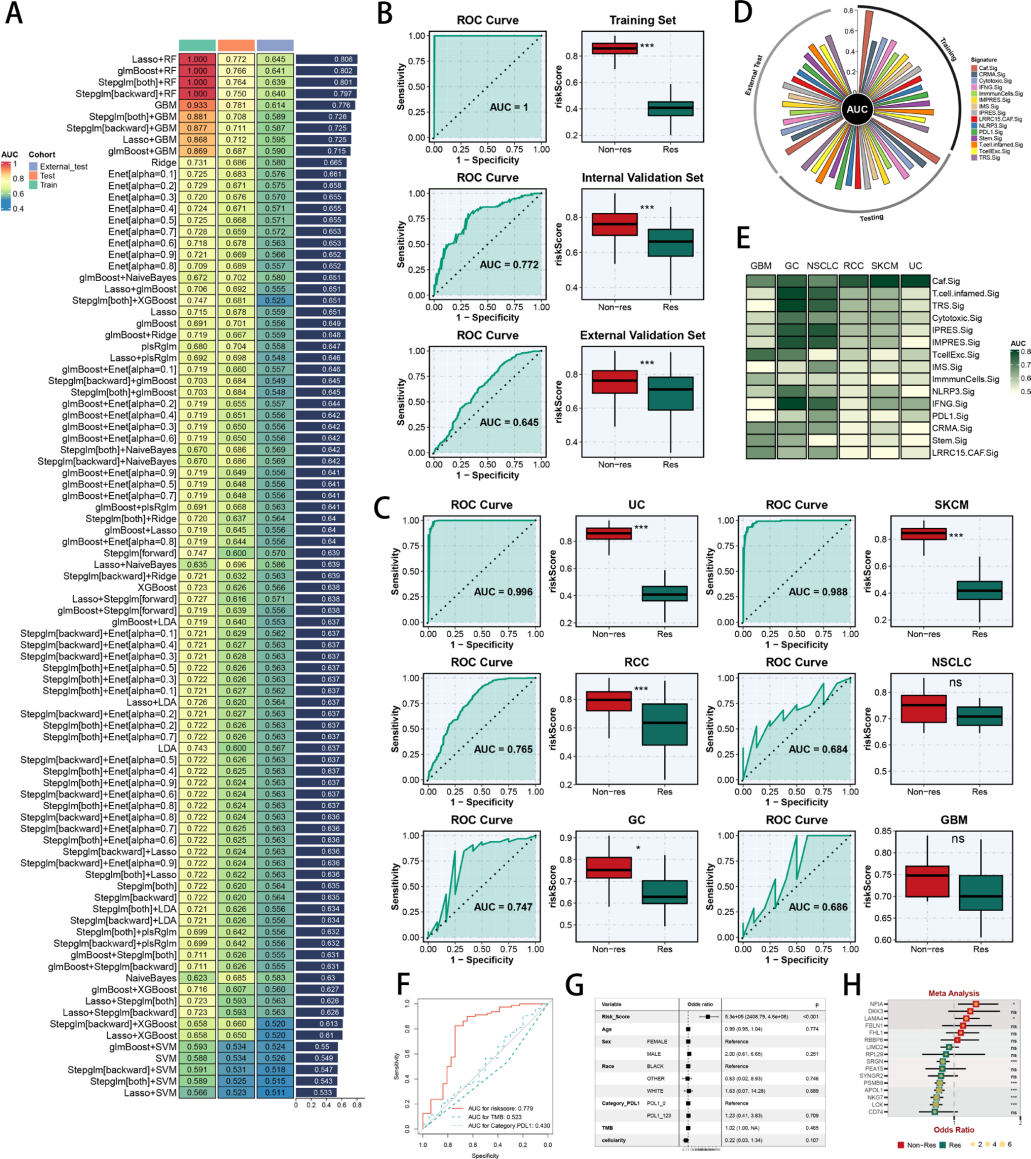
Development and validation of the Caf.Sig model to predict ICI response. (A) Construction of the Caf.Sig model using various machine learning combinations, with values in the heatmap representing the AUC of corresponding models for predicting ICI response; the bar graph on the right shows the average AUC across multiple datasets. (B) ROC curves of the Caf.Sig model to predict ICI response (left); as well as the distribution of Caf.Sig risk scores between responders and non‐responders (right), in the training, internal validation and external validation cohorts. (C) ROC curves of the Caf.Sig model to predict ICI response (left); as well as the distribution of Caf.Sig risk scores between responders and non‐responders (right), in the UC, SKCM, RCC, NSCLC, GC and GBM cohorts. (D) Comparison of the AUCs among the Caf.Sig model and other published signatures in the training, internal validation and external validation cohorts (Bar of AUC > 0.8 was not plotted). (E) Comparison of the AUCs among the Caf.Sig model and other published signatures in the UC, SKCM, RCC, NSCLC, GC and GBM cohorts. (F) Comparing Caf.Sig risk scores with TMB and PDL‐1 based on ROC Curves in the IMmotion150 cohort. (G) Multivariate logistic regression analysis in the IMmotion150 cohort. (H) Meta‐analysis of odds ratio of model genes from the Caf.Sig model in the training, internal validation and external validation cohorts. *p < 0.05; **p < 0.01; ***p < 0.001; ****p < 0.0001.

The above‐mentioned analysis results highlighted that fibroblasts could possibly modulate the immune response, so we further analysed the marker genes of fibroblasts. To delve deeper into the predictive potential of these genes, we assembled RNA‐seq and clinical data from 14 ICI treatment cohorts encompassing diverse solid tumours. As mentioned above, we divided those cohorts into training, internal test and external test cohorts, to use marker genes of fibroblasts in feature selection and model development. Utilising 113 predictive ML combinations and LOOCV framework, we refined a highly significant model and benchmarked it against previously established ICI‐related signatures (Figure 2A). The most powerful signature was developed by Lasso in feature selection and constructed by RF in model development, with the highest average AUC (0.806) across three cohorts (Figure 2A). AUC of the best Caf.Sig Model was illustrated in every cohort, while the Caf.Sig score calculated by the best Caf.Sig Model indicated that the non‐responders had significantly higher Caf.Sig.Score (Figure 2B). In combined datasets of every cancer type, we also found the superior AUC values of the Caf.Sig Model, as well as the significantly higher Caf.Sig score in non‐responders (Figure 2C). Compared with previously established ICI‐related signatures, the Caf.Sig Model exhibited significantly higher AUC values in the IMmotion dataset, suggesting its outstanding performances of predicting ICI response in RCC patients (Figure S2A). Meanwhile, comparative analysis revealed that the Caf.Sig Model exhibited superior predictive performance across the training, internal validation and external validation cohorts (Figure 2D), as well as in combined datasets of every kind of cancer type (Figure 2E). We then utilised a confusion matrix to show a fine precision of the Caf.Sig Model (Figure S2B). Leveraging the comprehensive clinical data available in the IMmotion150, IMmotion151, Mariathasan, Braun and Riaz cohorts, we conducted ROC curves to assess the Caf.Sig Model's prediction performances with PD‐L1 expression and TMB. The Caf.Sig Model continuously outperformed both PD‐L1 and TMB in multiple cohorts (Figure 2F, Figure S2C). Multivariate logistic regression analysis further identified the Caf.Sig risk score as an independent predictor of clinic response to ICI (all *p* < 0.05) (Figure 2G, Figure S2D). We calculated AUC of the Caf.Sig Model, several clinical variables and the nomogram model in the Braun cohort, indicating the superior capability of the Caf.Sig Model (Figure S2E). Calibration curves demenstrated well alignments between the Caf.Sig Model's predicted probability and observed probability (Figure S2F). DCA curves in the Braun cohort displayed the well clinic benefit of the Caf.Sig Model, outperforming other clinical predictors (Figure S2G). We then utilised the nomogram to incorporate several clinical factors and the Caf.Sig Model, helping clinical decision‐making (Figure S2H). Moreover, utilising univariate logistic analysis to explore the predictive value of model genes in three cohorts, we illustrated the odds ratio of every model gene of the Caf.Sig Model through meta‐analysis, which revealed the risky and protective model genes of ICI non‐response (Figure 2H). Furthermore, comprehensive pan‐cancer analysis of Caf.Sig risk score and immune‐related genes, immune cell abundance, TMB and MSI status revealed that the model was remarkably linked to the immune and mutational characteristics of the tumour microenvironment (Figure S2I–K).

### 3.3 CAF Heterogeneity and Role of iCAF in ICI Response

**FIGURE 3:**
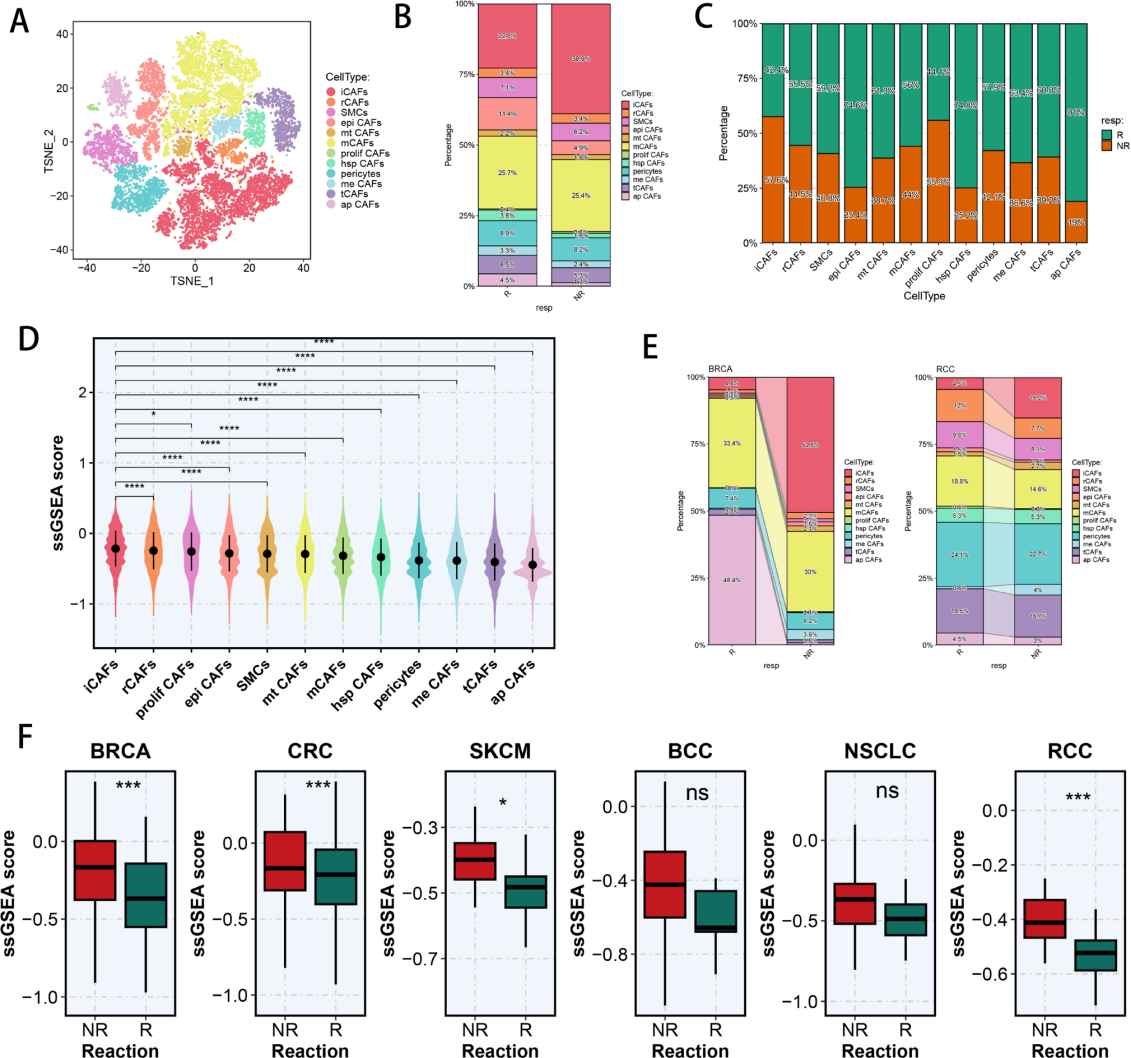
Pan‐cancer CAF heterogeneity and different Caf.Sig.Score in CAF subpopulations between responders and non‐responders. (A) Colour‐coded tSNE plot of 10 CAF subgroups, pericytes and smooth muscle cells. (B) The proportion of CAF subgroups in ICI responders and non‐responders. (C) The proportions of ICI responders and non‐responders in each CAF subgroup. (D) Caf.Sig.Score calculated by ssGSEA algorithm of each CAF subset. (E) The proportion of each CAF subgroup in ICI responders and non‐responders of BRCA and RCC. (F) The Caf.Sig.Score of iCAFs between ICI responders and non‐responders of six cancer types. ICI, immune checkpoint inhibitor; tSNE, T‐distributed stochastic neighbour embedding. *p < 0.05; **p < 0.01; ***p < 0.001; ****p < 0.0001.

Subsequently, we analysed the heterogeneity of CAFs in the pan‐cancer ICI treatment context and finally identified 10 CAF subtypes, pericytes and smooth muscle cells (Figure 3A). Based on a literature review, we identified the CAF subtype that remarkably expresses components of the complement system (C3, C7 and CFD, Figure S3A), annotating it as iCAF [72]. We also found a CAF subtype with over‐expressed ECM remodelling genes (COL11A1, CTHRC1 and POSTN, Figure S3A), annotating it as matrix CAF (mCAF) [73]. Meanwhile, a CAF subtype that over‐expressed MHC‐II‐associated antigen presentation genes (HLA‐DRA, HLA‐DRB1 and CD74, Figure S3A) was named as antigen presenting CAF (ap CAF) [74]. Besides, a CAF subtype that was enriched in glycolytic processes and ATP generation from ADP in GO BP terms (Figure S3B), naming it as metabolic CAF (meta CAF) [75]. Subsequently, a CAF subtype that over‐expressed cell cycle‐related genes (CENPF, STMN1 and TUBA1B, Figure S3A) was named as proliferative CAF (prolif CAF) [76]. After precise annotation, we found that the proportion of iCAFs was distinctly higher in non‐responders, suggesting its possibly important role in ICI treatment response (Figure 3B). As well, we found a greater number and percentage of iCAFs and prolif CAFs among non‐responders (Figure 3C). Therefore, we calculated the Caf.Sig.Score of every CAF subtype using the ssGSEA algorithm with model genes of the Caf.Sig model, which revealed that iCAFs have the highest M.Sig.Score, indicating the possibly unfavourable function of iCAFs for ICI therapy effectiveness (Figure 3D). We also analysed the expression profiles of model genes in the Caf.Sig model and revealed that the expression level of FBLN1 was significantly higher in iCAFs than in others (Figure S3C). Moreover, six other types of scRNA‐seq scoring algorithms also validated the top Caf.Sig.Score of iCAFs in pan‐cancer ICI treatment landscapes (Figure S3D). Then we compared the proportion of CAF subpopulations of responders and non‐responders in BRCA and RCC, suggesting a greater proportion of iCAFs in non‐responders (Figure 3E). Besides, the Caf.Sig.Score of iCAFs is relatively higher in non‐responders than in responders across every cancer type, speculating that iCAFs could play a crucial role in the failure of ICI treatment (Figure 3F). To further explore the associations between each CAF subtype and ICI response, we analysed immune evasion and immune checkpoint genes among each CAF subpopulation, revealing the low expression ratio of PDCD1, LAG3, PPARG and CD274 across every CAF subpopulation (Figure S3E). Subsequently, we examined the TF activities of CAF subpopulations and found that the expressions of HOXD4 and DBX2 were high in iCAFs (Figure S3F).

### 3.4 Interaction of iCAFs With CD8 ^+^ Tex Cells to Affect ICI Efficacy

**FIGURE 4:**
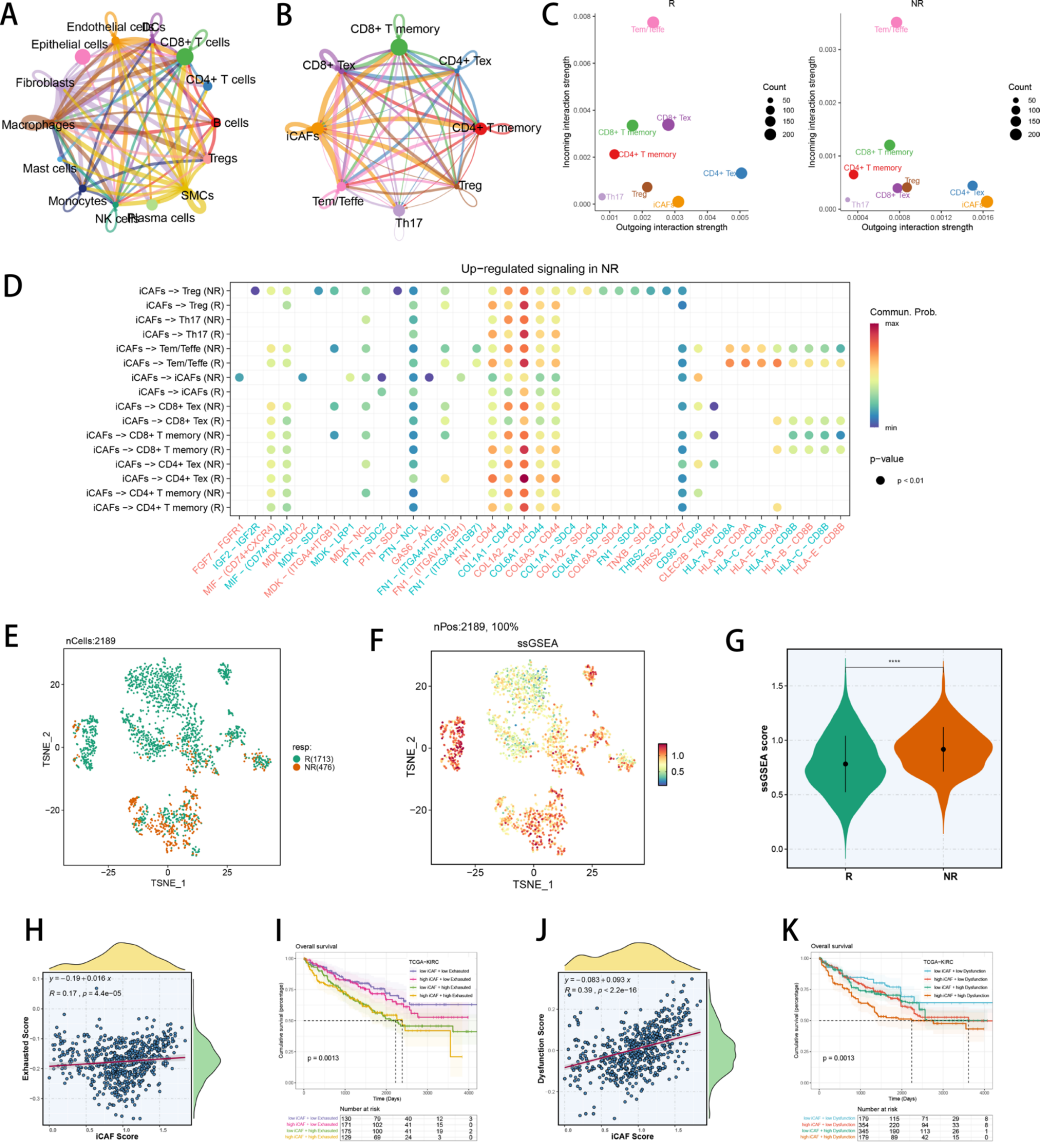
The communication between iCAFs and CD8^+^ Tex cells could affect ICI response. (A) Cell–cell interactions among major cell types. (B) Cell–cell interactions among iCAFs and different T cell subpopulations. (C) The relationship between differential outgoing interactions and incoming interaction strength for iCAFs and different T cell subpopulations. (D) Ligands and receptors for signal communication among iCAFs and different T cell subpopulations. (E) Colour‐coded UMAP plot of responders and non‐responders in CD8^+^ Tex cells. (F) Exhausted score of CD8^+^ Tex cells in UMAP. (G) Exhausted score of CD8^+^ Tex cells between ICI responders and non‐responders. (H) Correlation of Exhausted score with iCAF score in TCGA‐KIRC cohort. (I) Survival analysis of distinct Exhausted score and iCAF score in TCGA‐KIRC cohort. (J) Correlation of Dysfunction score with iCAF score in TCGA‐KIRC cohort. (K) Survival analysis of distinct Dysfunction score and iCAF score in TCGA‐KIRC cohort. ICI, immune checkpoint inhibitor; KIRC, kidney renal clear cell carcinoma; TCGA, The Cancer Genome Atlas; UMAP, Uniform Manifold Approximation and Projection. *p < 0.05; **p < 0.01; ***p < 0.001; ****p < 0.0001.

To deeply investigate the mechanisms of iCAFs affecting the effectiveness of ICI in the pan‐cancer landscape, we analysed interplays among the major cell types by exploring distinct cellular communications, demonstrating that fibroblasts and CD8^+^ T cells engaged in most intercellular communications (Figure 4A). We observed that CD8^+^ T cells displayed higher incoming communication intensity in responders, while fibroblasts showed the highest outgoing communication intensity both in responders and non‐responders (Figure S4A). Subsequently, we conducted dimension reduction clustering on T cells in pan‐cancer data and identified 12 T cell subpopulations (Figure S4B). Therefore, we performed intercellular communication analysis between T cell subpopulations and iCAFs, indicating that iCAFs and CD8^+^ T exhausted (Tex) cells engaged in a large proportion of intercellular chatting (Figure 4B). Then we found that in non‐responders, CD8^+^ Tex cells showed more incoming communication intensity, while iCAFs showed more outgoing communication intensity (Figure 4C). Moreover, the interactions of COL1A2‐CD44 on iCAFs was powerful, which could affect ICI effectiveness by communicating with CD8^+^ Tex cells and T effector memory/T effector (Tem/Teffe) cells (Figure 4D). In CD8^+^ Tex cells, we discovered several DEGs between responders and non‐responders, including GZMK, NKG7 and CTLA4 (Figure S4C,D). To explore the function of CD8^+^ Tex cells in ICI efficacy, we utilised UMAP to exhibit their distributions in responders and non‐responders (Figure 4E), and computed the exhausted score using ssGSEA (Figure 4F). We then observed that responders showed smaller exhausted scores than non‐responders in CD8^+^ Tex cells (Figure 4G). Meanwhile, we explored the effector scores of Tem/Teffe cells in responders and non‐responders, revealing that responders owned higher effector scores than non‐responders (Figure S4E–G). Above all, we speculated that CD8^+^ Tex could cause non‐response to ICI therapy, whereas Tem/Teffe were helpful for ICI responsiveness, indicating that response to ICI could be associated with the interactions of iCAFs with CD8^+^ Tex. Subsequently, we further explored the correlations of iCAFs and CD8^+^ Tex in the RCC specific context, and computed the exhausted score and iCAF score in the TCGA‐KIRC cohort using ssGSEA. Dysfunction scores downloaded from UCSC Xena were also incorporated for analysis. Intriguingly, we observed that the exhausted scores, as well as dysfunction scores, were significantly positively related to the iCAF scores in the TCGA‐KIRC cohort (Figure 4H,J). Moreover, we explored the differential immune cell abundances and immune functions in patients with high or low iCAF scores in the TCGA‐KIRC cohort, revealing the disparate immune infiltrations distinguished by iCAF scores (Figure S4H,I). Then we separated TCGA‐KIRC patients into four groups based on the median exhausted score, as well as the median dysfunction score, and the median iCAF score, revealing that patients with high exhausted scores and high iCAF scores owned the poorest prognosis (*p* < 0.05), as well as patients with high dysfunction scores and high iCAF scores (*p* < 0.05, Figure 4I,K). The aforementioned results demonstrated that iCAFs could possibly communicate with CD8^+^ Tex cells to influence the responsiveness of ICI therapy and the prognosis of KIRC patients.

### 3.5 Spatial Distribution Characteristics of ICI Treated Patients

**FIGURE 5:**
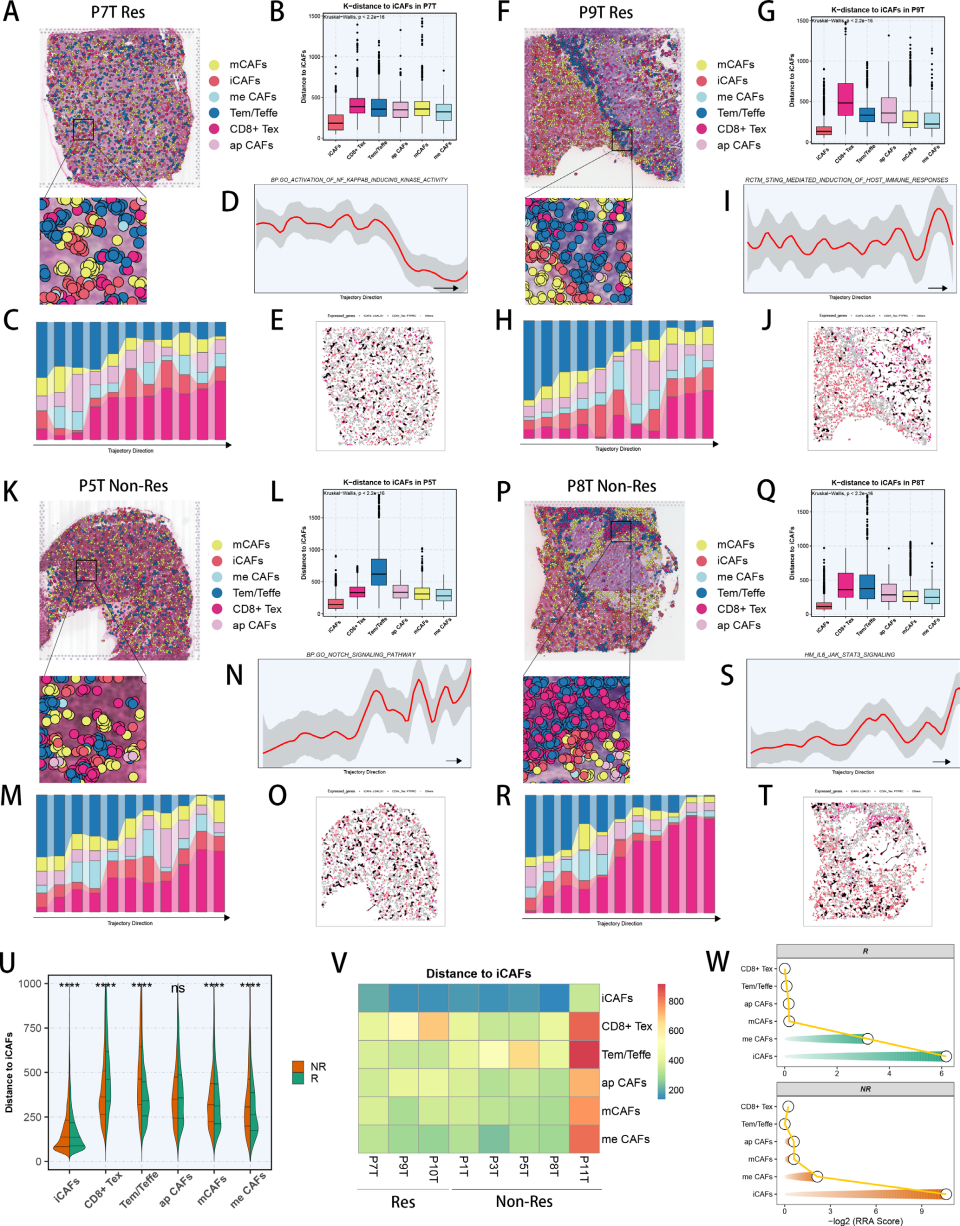
Spatial distribution characteristics of CAF subpopulations and T cell subpopulations. P7T and P9T were responders, while P5T and P8T were non‐responders. (A, F, K, P) Spatial cell charting of CAF subpopulations and T cell subpopulations in P7T (A), P9T (F), P5T (K) and P8T (P) using CellTrek. (B, G, L, Q) Spatial k‐distance to iCAFs among CAF subpopulations and T cell subpopulations in P7T (B), P9T (G), P5T (L) and P8T (Q). (C, H, M, R) Changes in cell proportions of six cell subtypes along the trajectory direction of Tem/Teffe shifting to CD8^+^ Tex in P7T (C), P9T (H), P5T (M) and P8T (R). (D, I, N, S) Changes in the specific pathway activity of six cell subtypes along the trajectory direction of Tem/Teffe shifting to CD8^+^ Tex in P7T (D), P9T (I), P5T (N) and P8T (S). (E, J, O, T) Spatial distribution of the LGALS1‐PTPRC interaction in P7T (E), P9T (J), P5T (O) and P8T (T). (U) Violin plot showing the spatial k‐distance to iCAFs in responders and non‐responders. (V) Heatmap showing the average k‐distance from different cell types to iCAFs in each tissue slice. The columns were scaled. (W) Integrated ranking of cell types based on proximity to iCAFs using RRA algorithm in responders and non‐responders. The smaller the RRA score of a certain cell type, the closer it is to iCAFs.

To further explore the spatial distribution characteristics of CAF subpopulations and T cell subpopulations in ICI responders and non‐responders, we downloaded scRNA‐seq and ST‐seq data of eight HCC patients receiving ICI therapy (non‐responders, *n* = 5; responders, *n* = 3) [53]. After quality control (Figure S5A), according to the unbiased clustering and spot features, spots of tumour sections were annotated as the literature's annotations (Figure S5B). We then utilised CellTrek to locate individual cells straightly to the corresponding spatial spots in tissue sections by incorporating scRNA‐seq and ST‐seq data from the same HCC patient [55]. Apart from traditional ST‐seq deconvolution algorithms, this technique transfers ST‐seq coordinates to single cells, setting the resolution at single cell level and reconstructing the spatially single cell atlases (Figure 5A,F,K,P). The CellTrek mapping analysis successfully demonstrated the spatial locations of mCAFs, iCAFs, me CAFs, ap CAFs, CD8^+^ Tex cells and Tem/Teffe cells in HCC tumour sections, which indicated abundant infiltrations of iCAFs, CD8^+^ Tex cells and Tem/Teffe cells in the tumour microenvironment (Figure 5A,F,K,P). Therefore, we analysed the spatial k‐distance among CAF subpopulations and T cell subpopulations in every tumour section, indicating that iCAFs exhibited closer distance with Tem/Teffe cells in responders, while iCAFs would exhibit closer distance with CD8^+^ Tex cells in non‐responders (Figure 5B,G,L,Q). To explore spatial expression dynamics from high‐density Tem/Teffe to CD8^+^ Tex regions, we identified high‐density regions of various cell types and performed spatial trajectory analysis in HCC sections. We firstly utilised ‘UCell’ algorithm to perform gene set enrichment analysis in ST‐seq data based on marker genes of every cell subpopulation, and figured out high‐density regions of every cell subpopulation (Figure S5C). Then, we drew the possible spatial trajectories of Tem/Teffe shifting to CD8^+^ Tex based on the high‐density cell regions (Figure S5D). With these spatial trajectories, we performed spatial trajectory analysis and revealed a progressive shift in proportions of Tem/Teffe and CD8^+^ Tex along the trajectory (Figure 5C,H,M,R), accompanied by variable presences of some signalling pathways. NF kappa B signalling pathway showed a decreasing trend in ICI responders, while displaying an unstable trend in ICI non‐responders (Figure 5D, Figure S6A), indicating the possible activation of NF kappa B signalling pathway in Tem/Teffe cells of responders. Besides, the activities of STING signalling pathway (Figure 5I, Figure S6B), NOTCH signalling pathway (Figure 5N, Figure S6C) and IL6_JAK_STAT3 signalling pathway (Figure 5S, Figure S6D) were unstable in responders, while their activities showed a slightly increasing trend in non‐responders, demonstrating the probable activations of these signalling pathways in CD8^+^ Tex of non‐responders. Moreover, we conducted Spatalk analysis to reveal the spatial communication patterns of iCAFs and CD8^+^ Tex in HCC sections. Given that iCAFs could cause apoptosis in CD8^+^ T cells and decrease anti‐cancer immunity through interplaying with CD8^+^ T cells PTPRC receptors by Galectin‐1 (LGALS1) based on literature review [77, 78], we explored the ligand‐receptor interactions (LRIs) of LGALS1‐PTPRC (Figure 5E,J,O,T), and found that the interplay distances of LGALS1‐PTPRC LRI were closer in non‐responders than in responders (Figure S6E). Other LRIs associated with immunosuppression were also analysed in tumour sections from responders and non‐responders, and the RRA algorithm was employed to compute a comprehensive ranking of LRIs in responders and non‐responders, respectively (Figure S6F). We discovered that iCAFs could reduce the activation and proliferation of CD8^+^ T cells via the MIF‐CXCR4 interaction [79] (Figure S6F). Additionally, iCAFs may secrete TGF‐β1 to further suppress the activation and proliferation of CD8^+^ T cells [80] (Figure S6F). Afterwards, combining cell‐to‐cell k‐distance in all tumour sections, we analysed the spatial k‐distance to iCAFs in responders and non‐responders, suggesting that the distance between iCAFs and Tem/Teffe cells was closer in responders, whereas the distance between iCAFs and CD8^+^ Tex cells was closer in non‐responders (Figure 5U). The heatmap of k‐distance to iCAFs in each section also verified the aforementioned results (Figure 5V). Besides, we utilised the RRA algorithm to acquire an overall ranking of k‐distance to iCAFs, revealing that iCAFs located closer to Tem/Teffe cells in responders, while iCAFs stayed closer to CD8^+^ Tex cells in non‐responders (Figure 5W). Above all, the ST‐seq analysis at spatially single cell resolution demonstrated that cellular distance between iCAFs and CD8^+^ Tex cells might play a critical role in response to ICI treatment for tumour patients.

### 3.6 Identification of GSN With Possibly Critical Functions in ICI Therapy

**FIGURE 6:**
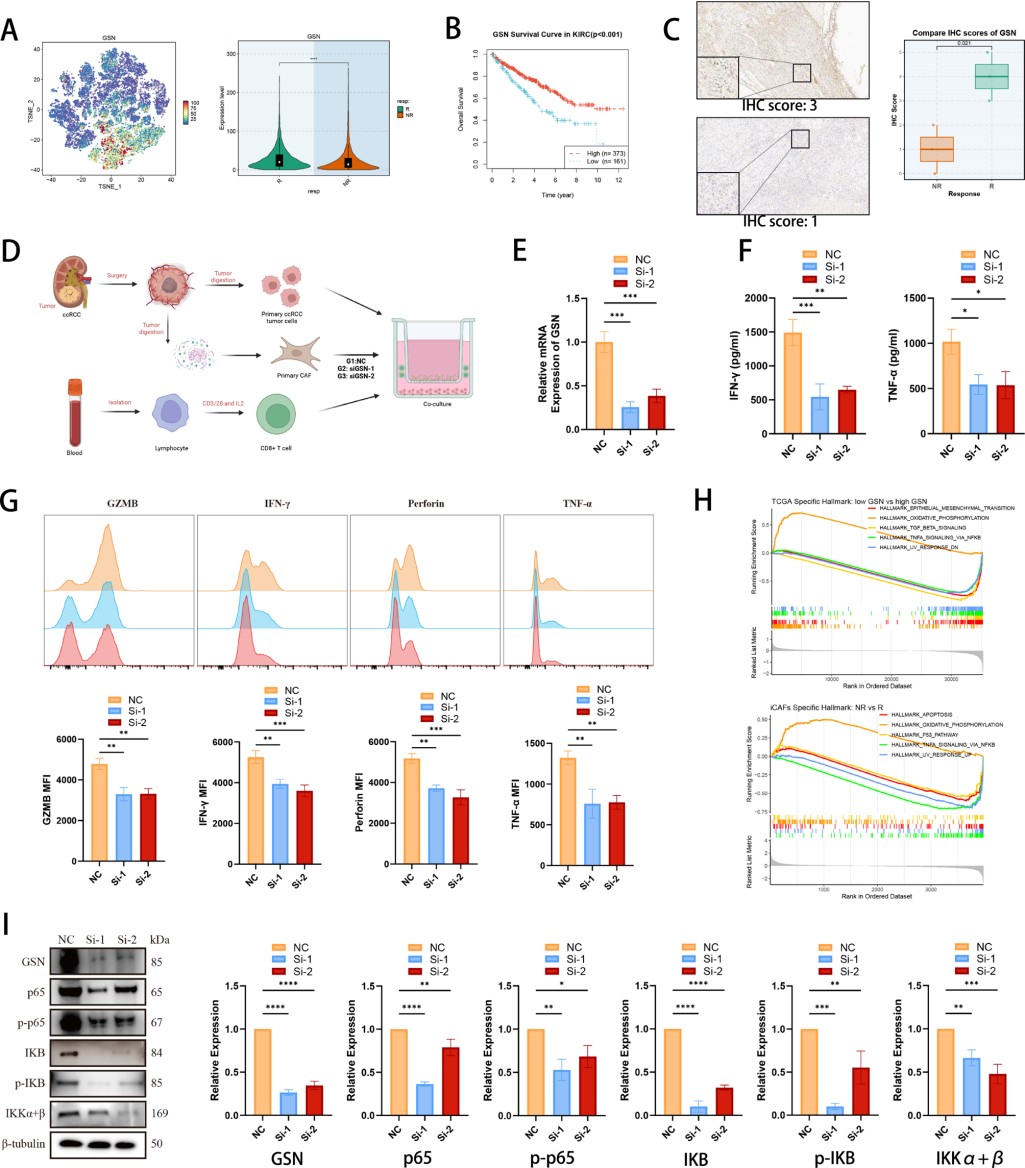
Silencing of GSN in CAFs inhibits intra‐tumoral CD8^+^ T cells function and drives them towards a dysfunctional state. (A) tSNE plot of iCAF marker gene GSN, and expression of GSN in iCAFs from responders and non‐responders. (B) Survival analysis of GSN (in terms of OS) in TCGA‐KIRC cohort. (C) Immunohistochemistry (IHC) staining in tumour samples of three non‐responders and three responders. (D) Workflow of the co‐culture system with primary CAFs (NC, siGSN‐1 and siGSN‐2), primary ccRCC tumour cells and CD8^+^ T cells. (E) qRT‐PCR analysis of GSN mRNA in three groups (NC, siGSN‐1 and siGSN‐2). (F) ELISA analysis of the IFN‐γ and TNF‐α levels in supernatant from the co‐culture system. (G) Flow cytometry analysis of IFN‐γ, TNF‐α, GZMB and Perforin in CD8^+^ T cells isolated from the co‐culture system. (H) GSEA analysis in TCGA‐KIRC cohort (low GSN vs. high GSN), as well as in pan‐cancer scRNA‐seq landscape of ICI therapy (non‐responders vs. responders). (I) Representative western blot of GSN, p65, p‐p65, IKB, p‐IKB and IKKα + β protein expression levels in the co‐culture system. *p < 0.05; **p < 0.01; ***p < 0.001; ****p < 0.0001.

Given that iCAFs could be of vital importance in ICI efficacy based on our bioinformatics analysis, we analysed the marker gene of iCAFs and sought to explore its underlying mechanisms affecting ICI response. Intriguingly, we found that the iCAF marker gene Gelsolin (GSN), with favourable prognosis value in ccRCC (Figure S7F), was up‐regulated in not only iCAFs from responders in the pan‐cancer landscape (Figure 6A), but also iCAFs from responders in the RCC cohort (Figure S7A), suggesting its possibly favourable function in the ICI response of RCC. Survival analysis further revealed that in the TCGA‐KIRC cohort, patients with high GSN expressions would have better overall survival (OS, *p* < 0.001, Figure 6B), as well as better progression‐free survival (PFS, *p* = 0.001, Figure S7B). Moreover, due to the vital function of CD8^+^ T cells in anti‐tumour immunity, we explored the prognostic value of GSN in patients with high or low CD8^+^ T cell infiltrations (separated by CD8A expressions) in the TCGA‐KIRC cohort. Interestingly, although ccRCC patients could not benefit from CD8^+^ T cell infiltrations, patients with high GSN expressions and high CD8^+^ T cell infiltrations showed the longest OS (*p* = 0.03, Figure S7C). Subsequently, we analysed the predictive value of GSN in ICI response prediction. Immunohistochemistry (IHC) staining was performed in tumour samples of three non‐responders and three responders from our hospital, which revealed that GSN was expressed significantly higher in responders than in non‐responders (Figure 6C). We then investigated the predictive value (in terms of AUC) of GSN in two RCC ICI cohorts (Figure S7D), as well as in pan‐cancer ICI cohorts (Figure S7E), revealing its well performances in predicting ICI response. Besides, we performed pan‐cancer analysis to discover the heterogeneity of GSN expression in tumoral and normal tissues, as well as its prognostic value in the pan‐cancer landscape (Figure S7F).

### 3.7 Downregulation of GSN on CAFs Drives CD8 ^+^ T Cells Towards a Dysfunctional State in RCC

Based on bioinformatics analysis, we could speculate that GSN played a favourable role in prognosis of RCC patients, and RCC patients with high GSN expression would probably respond to immunotherapy due to well cytotoxicity of CD8^+^ T cells. To investigate whether downregulation of GSN could decrease the cytotoxicity of CD8^+^ T cells in vitro, we isolated primary CAFs and primary ccRCC tumour cells from tumour samples of HLA‐A2^+^ ccRCC patients, and we also isolated CD8^+^ T cells from PBMC of the same patient. The primary CAFs were categorised into three groups as NC, siGSN‐1 and siGSN‐2. To deeply explore the immune function of GSN in CAFs, an indirect co‐culture condition was constructed among primary CAFs (NC, siGSN‐1 and siGSN‐2), primary ccRCC tumour cells and CD8^+^ T cells (Figure 6D). Quantitative real‐time PCR (qRT‐PCR), as well as western blotting (WB) validated the successful silencing of GSN on CAFs (Figure 6E,I). Enzyme‐linked immunosorbent assay (ELISA) showed that downregulation of GSN caused decreased production of cytokines (Figure 6F). We then isolated CD8^+^ T cells from the co‐culture system, and utilised flow cytometry analysis to reveal that effector function of CD8^+^ T cells suffered a loss after silencing GSN in CAFs (Figure 6G, Figure S7G). Subsequently, we sought to investigated the underlying mechanisms of reduced effector function after downregulation of GSN in CAFs, so we performed GSEA analysis in TCGA‐KIRC cohort. Compared to patients with high GSN expression, patients with low GSN expression displayed a decreased enrichment of ‘TNF‐α signalling via NFκB’ (Figure 6H). As well, a reduced enrichment of ‘TNF‐α signalling via NFκB’ was also observed in iCAFs from non‐responders, compared to iCAFs from responders, in pan‐cancer scRNA‐seq landscape of ICI therapy (Figure 6H). Therefore, WB analysis was conducted in cells from the co‐culture system, revealing that silencing of GSN in CAFs significantly reduced the protein levels of p65, p‐p65, IKB, p‐IKB and IKKα + β (Figure 6I). Therefore, the aforementioned results suggested that GSN could activate the NFκB signalling pathway in the co‐culture system.

### 3.8 Upregulation of GSN in CAFs Enhances the Efficacy of Immunotherapy in RCC Animal Model

**FIGURE 7:**
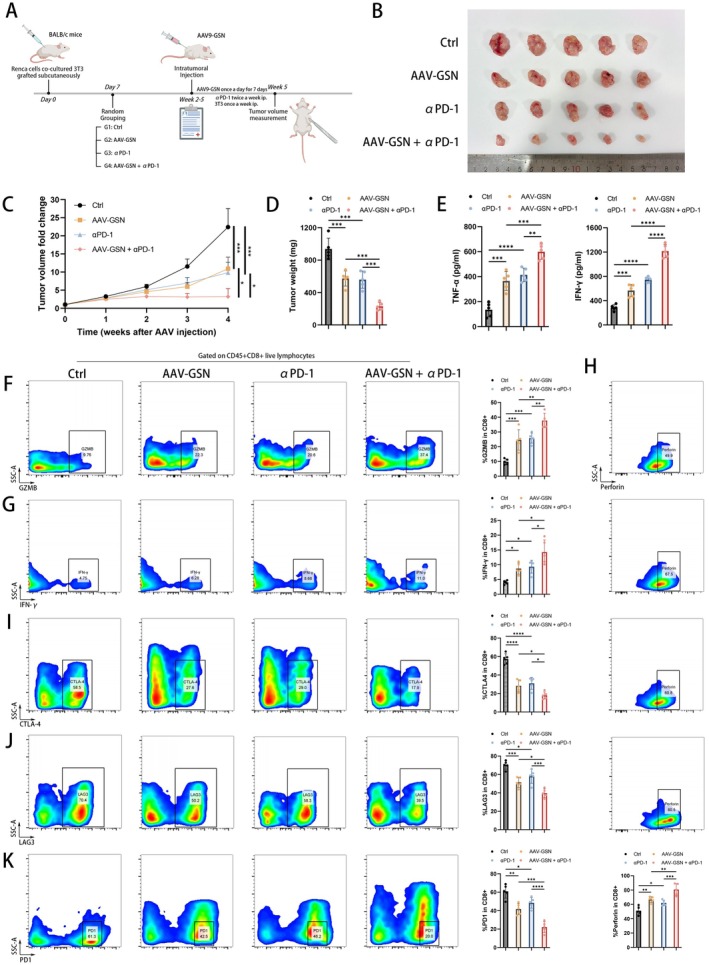
(A) The subcutaneously experimental design of mice. (B–D) The anti‐PD‐1 and AAV‐GSN combination therapy synergistically suppressed the growth of tumours in RCC subcutaneous tumour models (*n* = 5 per group). (B) tumour images, (C) tumour growth curves, (D) tumour weights. (E) ELISA analysis of the IFN‐γ and TNF‐α levels in serum samples from RCC subcutaneous tumour models with the indicated treatments. (F–H) Flow cytometry analysis of GZMB (F), IFN‐γ (G) and Perforin (H) in CD8^+^ T cells isolated from RCC subcutaneous tumour models with the indicated treatments. (I–K) Flow cytometry analysis of CTLA‐4 (I), LAG3 (J) and PD1 (K) in CD8^+^ T cells isolated from RCC subcutaneous tumour models with the indicated treatments. *p < 0.05; **p < 0.01; ***p < 0.001; ****p < 0.0001.

**FIGURE 8:**
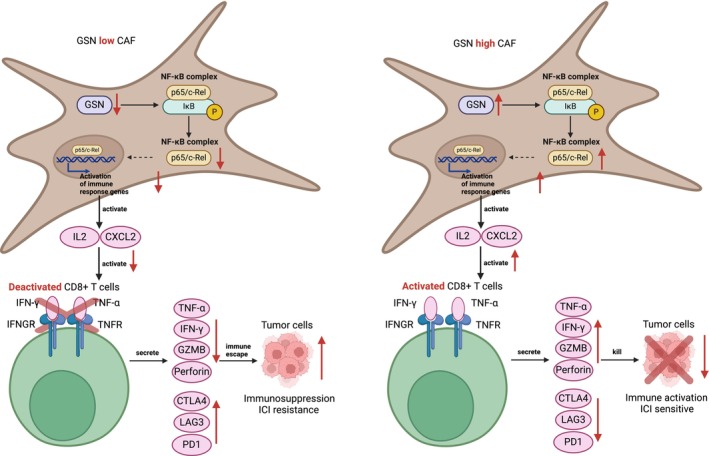
Schematic model depicting the key findings of this study. Silencing GSN in CAFs could inactivate NFκB signalling pathway, causing the reductions of pro‐immune cytokines and resulting in inactivated CD8^+^ T cells. The inactivated CD8^+^ T cells would secrete less cytokines and release more exhausted molecules, which finally led to immunosuppression and immune escape of ccRCC tumour cells.

According to the aforementioned results, we then investigated the feasibility of upregulating GSN in CAFs in vivo to enhance the therapeutic effectiveness of ICI. The Renca cells co‐cultured with 3T3 cells were grafted subcutaneously into the BALB/c mice in four groups (Ctrl, AAV‐GSN, αPD‐1 and AAV‐GSN + αPD‐1, *n* = 5), and GSN overexpression lentivirus was constructed to upregulate the GSN expression in CAFs, which were later injected into the subcutaneous tumour models (Figure 7A). Injection with AAV‐GSN into subcutaneous tumours effectively upregulated GSN mRNA expression in mouse tumour tissues (Figure S8). Subsequent analysis displayed that, despite both AAV‐GSN and anti‐PD‐1 monoclonal antibody being able to solely inhibit tumour growth, combining AAV‐GSN and PD‐1 blockade treatments could remarkably decrease tumour growth and tumour volume compared to therapy with AAV‐GSN or PD‐1 blockade alone (Figure 7B–D). Moreover, we conducted ELISA analysis to reveal that combining AAV‐GSN treatments and anti‐PD‐1 monoclonal antibody could remarkably enhance the effector function of CD8^+^ T cells (Figure 7E). Meanwhile, flow cytometry analysis also validated the significant improvement of the effector function of CD8^+^ T cells, as well as the reduction of exhaustion molecules in CD8^+^ T cells, after combinations of AAV‐GSN and anti‐PD‐1 treatment (Figure 7F–K). In summary, these results demonstrated that upregulation of GSN in CAFs could significantly increase the therapeutic efficacy of PD‐1 blockade and might be a potential therapy strategy to enhance ICI effectiveness in ccRCC. Taken together, we speculated that silencing GSN in CAFs could inactivate NFκB signalling pathway, causing the reductions of pro‐immune cytokines and resulting in inactivated CD8^+^ T cells. The inactivated CD8^+^ T cells would secrete less cytokines and release more exhausted molecules, which finally led to immunosuppression and immune escape of ccRCC tumour cells (Figure 8).

## 4 Discussion

Nowadays, ICI therapies have emerged as first‐line and novel adjuvant therapeutic methods for the majority of early‐stage and advanced cancers. Previously, a pan‐cancer scRNA‐seq and ST study has highlighted a connection between iCAFs and ICI efficacy across various cancers, with CD8^+^ T cells often residing in iCAFs‐rich regions within the TME [30]. This association indicates that iCAFs could possibly suppress antitumor T‐cell immunity in solid tumours, correlating with poor prognosis and treatment failure. In our study, we analysed fibroblast‐associated gene expression in ICI responders and non‐responders. Using multiple ML techniques, we identified the Lasso + RF algorithm as optimal for constructing a Caf.Sig model to accurately predict ICI response across cancers. This integration is achieved through an ensemble learning framework, which combines the feature selection capabilities of Lasso with the predictive power of RF. Our experimental findings further suggest that GSN^+^ iCAFs, which are closely linked to CD8^+^ Tex cells, possibly contribute to immunotherapy failure in ccRCC.

Previous research has primarily focused on investigating the link between CAFs and clinic characteristics. Researchers have found that increased percentages of FAP + CAFs were associated with bigger tumours, synchronous metastases and tumour aggression based on multiplex immunofluorescence analysis [81]. Previous studies also revealed that the tumour‐associated mesenchymal stromal cell population in bone marrow stroma is correlated with poor PFS and OS for ccRCC patients with bone metastasis [82]. Additionally, many studies have demonstrated that CAF‐associated gene signatures and specific CAF subsets could considerably influence TME and immunotherapy efficacy [83, 84]. Recently, ML has served as a widely applied assistant in medical research [85], which utilises intricate algorithms to automatically handle expansive and complex information [86]. However, this approach has not been widely utilised in fibroblast‐related research in ccRCC, especially for predicting responses to ICI therapies. In our study, we utilised an integrated ML framework to construct a Caf.Sig model based on the expression profiles of fibroblast marker genes, aiming to precisely predict the response to immunotherapy across multiple cancers. A total of 113 predictive algorithms were incorporated in the training cohort via LOOCV framework. Further validations in internal and external validation datasets disclosed that the most effective algorithm was Lasso in feature selection and RF in model construction. The resilience of this integrated approach resides in its capability to integrate multiple ML algorithms. This integration leads to the creation of models with superior predictive capabilities. By decreasing the dimensionality of a large number of variables, this approach streamlines the model for practical and translational applications. The effectiveness of the Caf.Sig model was verified using the confusion matrix, ROC curves, AUC values, calibration curves and DCA curves. These evaluation metrics highlight its superiority over other clinical variables and suggest its great potential in clinical decision‐making.

Compared to the previously published signatures used to predict immunotherapy efficacy, the AUC of the Caf.Sig model was better in the overall cohort, as well as in the individual subgroups, suggesting its broad applicability across various cancer types. Most model genes in the Caf.Sig model are not part of the 14 immune gene signatures mentioned earlier. Nuclear Factor I A (NFIA) and Laminin Subunit Alpha 4 (LAMA4), two critical model genes of the Caf.Sig model, were negatively associated with the response to immunotherapy based on meta‐analysis of their OR values in three cohorts. Researchers have found that NFIA promotes the growth and migration of glioblastoma by negatively regulating p53, p21 and PAI1 [87]. Previous studies showed that LAMA4 could affect tumour cell behaviour by promoting migration and invasion, and induce tumour progression by regulating the composition and structure of the ECM [88]. In this case, to better investigate how the Caf.Sig model predicts responses to immunotherapy, we categorised fibroblasts into various subgroups and examined their disparities between responders and non‐responders and their association with the Caf.Sig score. Notably, we found that iCAFs, which are involved in the development of an immunosuppressive microenvironment through interactions with macrophages and CD8^+^ T cells, are co‐localised with CD8^+^ T cells in situ across various cancer types [30]. Moreover, we identified that the iCAF subset constituted the largest fraction of CAFs from non‐responders, while it indeed exhibited the highest Caf.Sig score, which suggested that the iCAFs may exert the strongest immunosuppressive effects on ICI therapy. For other CAF subsets, a prior study on myofibroblastic CAFs (myCAF) confirmed that myCAFs, a CAF subset with epithelial mesenchymal plasticity, were strongly correlated with mesenchymal‐like ccRCC cells, which were both abundant in metastases and correlated with poor prognosis [84]. Moreover, previous findings also highlighted the crucial function of POSTN^+^ CAFs as potential immune response barriers in specific tumour spots, as well as the interactions between POSTN^+^ CAFs and SPP1^+^ macrophages, which the former recruited the latter and induced increasing SPP1 expression through the IL‐6/STAT3 signalling pathway [89]. However, there was still a gap in the research field of iCAFs in ICI therapy, especially for ccRCC, which prompts us to deeply investigate its biological functions and explore potential mechanisms in shaping the immunosuppressive microenvironment.

To better comprehend the function of the iCAF subset, we analysed the corresponding iCAF score in the ccRCC specific context via bulk‐seq analysis. We finally discovered the significant correlation of iCAF scores with the immune dysfunction scores and T cell exhaustion scores. Meanwhile, the results of scRNA‐seq analysis indicated a particularly striking disparity in the Caf.Sig scores between responders and non‐responders in the RCC cohort. The aforementioned results suggest that iCAFs may contribute significantly to immunotherapy resistance. Extensive research has shown that CAF subsets could modulate T cell function and shape the tumour‐fighting capacity of the immune microenvironment. Furthermore, our study highlighted the association between iCAFs and CD8^+^ Tex cells, reinforcing these findings through spatial trajectory analysis and spatial cell–cell communication inference. Previous research has revealed that the spatial arrangement of immune cells within the TME may influence biological processes in cancer patients. Some researchers observed that the distances between tumour cells and CD8^+^ PD‐1^+^ T cells in NSCLC would increase after immunotherapy [90]. Additionally, in lung tumour tissues, the proximity of CD8^+^ Treg cells to tumour cells correlates with patient prognosis, which means greater separation is related to a more favourable outcome [91]. In our spatial analysis, we revealed that the cellular distance between iCAFs and CD8^+^ Tex cells in non‐responders was closer than that in responders, indicating a higher probability of intercellular communication between iCAFs and CD8^+^ Tex cells in non‐responders. This could possibly drive immune evasion and T cell exhaustion due to chemokine and cytokine networks, immune checkpoint molecules, metabolic competition and chronic antigen stimulation [92]. Moreover, we found that the interplay distances of LGALS1‐PTPRC LRI were closer in non‐responders between iCAFs and CD8^+^ Tex cells, which could possibly drive T cell exhaustion and reduce anti‐tumour immunity.

Since our bioinformatics analysis suggested that iCAFs played a crucial role in ICI efficacy, we further examined their marker gene GSN (Gelsolin) and investigated its potential mechanisms influencing the ICI response in ccRCC. Previous studies have showed that GSN might affect the migration and invasion ability of tumour cells through governing the dynamic changes of the cytoskeleton in tumour cells, and GSN knockdown would cause severe DNA damage and promote apoptosis following radiotherapy [93]. In the ccRCC context, we discovered that elevated GSN expression was related to a better prognosis and an increased response to immunotherapy, which led us to focus on CD8^+^ T cell function and immunotherapy efficacy. Therefore, we performed in vitro co‐culture experiments of primary ccRCC tumour cells, CAFs and CD8^+^ T cells, which revealed that silencing of GSN in CAFs would cause a decrease in cytokines and inactivation of CD8^+^ T cells, possibly leading to resistance to immunotherapy. Moreover, we sought to explore the underlying mechanism of GSN affecting the ICI response. Based on GSEA analysis and WB validation, we proved the inactivation of the NF kappa B signalling pathway after GSN downregulation in CAFs and speculated that GSN in CAFs could probably influence the NF kappa B signalling pathway to affect the ICI response. In that case, inactivation of the NFκB signalling pathway could possibly result in decreased cytokine secretion and increased expression of exhaustion markers, ultimately causing immunosuppression and immune escape in ccRCC tumour cells. Subsequently, we performed in vivo experiments in the animal model to validate the influence of GSN upregulation on CD8^+^ T cell functions, which revealed an increase in cytokines and a reduction of exhaustion‐associated molecules after GSN upregulation in CAFs. Therefore, combining AAV‐GSN and anti‐PD‐1 treatment would emerge as a possible therapeutic approach to fight immunotherapy resistance and immune exhaustion of CD8^+^ T cells during ICI therapy.

Ultimately, our Caf.Sig model could be readily replicated using PCR detection techniques, making it suitable for broader clinical application. However, it is essential to acknowledge some setbacks in our study. First, our research was conducted retrospectively, with sequencing data and clinic information sourced from public databases. The absence of detailed treatment protocols, metastasis sites and recurrence data might impact our results. Second, the scRNA‐seq datasets employed for developing the Caf.Sig model encompassed merely nine types of cancers, which may introduce a degree of bias. Further validation of this model across a broader spectrum of cancer types is warranted, while integrating ST data of ccRCC to assess the spatial distribution of iCAFs in relation to immune cell infiltration, particularly CD8^+^ T cells, is also demanded in ccRCC‐specific research. Lastly, our current model establishment, which relies solely on transcriptome sequencing, would benefit remarkably from the integration of multi‐omics and multi‐modal data. The comprehensive integration enhances our comprehension of molecular mechanisms and physiological processes, thereby improving the accuracy and clinical utility of the predictive model. While our model performed well in internal and external validations, cross‐dataset reliability may vary due to technical biases between single‐cell and bulk platforms, let alone multi‐omics integration. Deep learning, a subset of ML, excels at independently identifying key classification features, a capability not easily achieved with previous ML methods that need manual feature selection. Thus, the adoption of advanced deep learning algorithms, combined with the abundant insights from multi‐omics and multi‐modal data integration, represents a powerful approach to advance personalised medicine for cancer patients.

## 5 Conclusion

In our research, we are the first to utilise large‐scale pan‐cancer scRNA‐seq and bulk RNA‐seq data of ICI therapy datasets to develop and validate the Caf.Sig model for predicting ICI response within ML algorithms. Additionally, we initially explored the underlying mechanisms in ccRCC by which the Caf.Sig model could forecast the ICI efficacy, specifically, by iCAFs communicating with CD8^+^ Tex cells. Upregulation of GSN on CAFs drives CD8^+^ T cells towards an effector state, and combining GSN overexpression with ICI treatment achieves optimal efficacy in ccRCC. Our research gives insights and strategies for solving the heterogeneity of ICI response and improving cancer immunotherapy.

## Author Contributions

S.L. and L.F. designed the research plan, performed bioinformatics analysis, performed experiments and wrote the manuscript. S.L., X.Z., H.F., K.H., M.C., M.L., H.L., Z.D., Y.C., W.L., Z.Z., J.C., B.G., T.S. and Z.F. performed the data collection, assembly of data and finalised the manuscript. A.Y., G.S., Y.P. and L.F. supervised the research progress and revised the manuscript. All authors contributed to the article and approved the submitted version.

## Ethics Statement

The studies involving human participants were reviewed and approved by the Institutional Ethics Committee for Clinical Research and Animal Trials Ethical of the First Affiliated Hospital of Sun Yat‐sen University. The patients/participants provided their written informed consent to participate in this study. The in vivo mouse experiments have been reviewed and approved by the Institutional Animal Care and Use Committee (IACUC), Sun Yat‐Sen University, and performed in accordance with the guidelines for the care and use of animals.

## Conflicts of Interest

The authors declare no conflicts of interest.

## Supporting information


**Figure S1.** (A) Different proportions of ICI treatment outcomes in nine scRNA‐seq cohorts. (B) Pseudotime DEGs identified by pseudotime analysis. (C) DEGs of fibroblasts in responders and non‐responders. (D) Distinction expression profiles of fibroblasts between responders and non‐responders.

**Figure S2.** (A) Comparison of the AUCs among the Caf.Sig model and other published signatures in IMmotion cohort. (B) Confusion matrix of the Caf.Sig Model in the training, internal validation and external validation cohorts. (C) Comparing Caf.Sig risk scores with TMB and PDL‐1 based on ROC Curves in IMmotion151, Mariathasan, Braun and Riaz cohorts. (D) Multivariate logistic regression analysis in IMmotion151, Mariathasan, Braun and Riaz cohorts. (E) AUC of the Caf.Sig Model, several clinical variables and the nomogram model in the Braun cohort. (F) Calibration curves in the training, internal validation and external validation cohorts. (G) DCA curves of the Caf.Sig Model, several clinical variables and the nomogram model in the Braun cohort. (H) The nomogram model in the Braun cohort. (I) Pan‐cancer analysis of associations among the Caf.Sig risk score and the expressions of immune‐related genes. (J) Pan‐cancer analysis of associations among the Caf.Sig risk score and various immune cell abundances. (K) Pan‐cancer analysis of associations among the Caf.Sig risk score and tumour mutational burden (TMB), and microsatellite instability (MSI) status.

**Figure S3.** (A) Visualising marker genes of each CAF subtype by dotplot. (B) Visualising marker genes of each CAF subtype by heatmap, as well as enrichment analysis results of each CAF subtype by GO and KEGG. (C) Expression of model genes from the Caf.Sig model in CAF subpopulations. (D) Utilising other six types of scRNA‐seq scoring algorithms (AUCell, Ucell, GSVA, singscore, AddModuleScore and PercentageFeatureSet) to validate the top Caf.Sig.Score of iCAFs. (E) Comparison of immune evasion and immune checkpoint genes among CAF subclusters. (F) Comparison of transcription factor activities among CAF subclusters.

**Figure S4.** (A) The relationship between differential outgoing interactions and incoming interaction strength for major cell types in responders and non‐responders. (B) Colour‐coded UMAP plot of T cell subgroups. (C) Distinction expression profiles of CD8^+^ Tex cells between responders and non‐responders. (D) DEGs of CD8^+^ Tex cells between responders and non‐responders. (E) Colour‐coded UMAP plot of responders and non‐responders in Tem/Teffe cells. (F) Effector score of Tem/Teffe cells in UMAP. (G) Comparison of effector score of Tem/Teffe cells between responders and non‐responders. (H) Differential immune cell abundances in patients with high or low iCAF scores in TCGA‐KIRC cohort. (I) Disparate immune function levels in patients with high or low iCAF scores in TCGA‐KIRC cohort.

**Figure S5.** (A) Violin plots show the quality control feature of percentage of mitochondrial genes in each patient. (B) Unbiased clustering of ST spots and cell types of each cluster in each patient. (C) High‐density regions of every cell subpopulation in each tumour section. (D) Spatial trajectory from high‐density areas of Tem/Teffe to high‐density areas of CD8^+^ Tex in four tumour sections.

**Figure S6.** (A) Changes in NF kappa B pathway activity of six cell subtypes along the trajectory direction of Tem/Teffe shifting to CD8^+^ Tex in three tumour sections. (B) Changes in STING pathway activity of six cell subtypes along the trajectory direction of Tem/Teffe shifting to CD8^+^ Tex in three tumour sections. (C) Changes in NOTCH pathway activity of six cell subtypes along the trajectory direction of Tem/Teffe shifting to CD8^+^ Tex in three tumour sections. (D) Changes in IL6_JAK_STAT3 pathway activity of six cell subtypes along the trajectory direction of Tem/Teffe shifting to CD8^+^ Tex in three tumour sections. (E) The interplay distances of LGALS1‐PTPRC in non‐responders and responders. (F) Integrated ranking of ligand‐receptor interactions based on interplay distances between iCAFs and CD8^+^ Tex using RRA algorithm in responders and non‐responders. The smaller the RRA score of a certain ligand‐receptor interaction, the closer it is between iCAFs and CD8^+^ Tex.

**Figure S7.** (A) Expression of GSN in iCAFs from responders and non‐responders in RCC scRNA‐seq cohort, as well as GSN expression in every CAF subpopulation in RCC scRNA‐seq cohort. (B) Survival analysis of GSN (in terms of PFS) in TCGA‐KIRC cohort. (C) Survival analysis of GSN and CD8A (in terms of OSS) in TCGA‐KIRC cohort. (D) AUC value of GSN to predict ICI response in two RCC cohorts. (E) AUC value of GSN to predict ICI response in pan‐cancer RNA‐seq cohorts. (F) Pan‐cancer RNA‐seq analysis of GSN expression in tumoral and normal samples, as well as its prognosis value. (G) Representative flow cytometry figure of IFN‐γ, TNF‐α, GZMB and Perforin in CD8^+^ T cells isolated from the co‐culture system.

**Figure S8.** qRT‐PCR analysis of GSN mRNA in tumours from four groups (Ctrl, AAV‐GSN, αPD‐1 and AAV‐GSN + αPD‐1).

## Data Availability

Publicly available datasets were analysed in this study. The names of the repositories and accession numbers can be found within the article/Supporting Information. Further inquiries can be directed to the corresponding author (L.F.).
