# Supplementary figures and images for: Deciphering the Immunomodulatory Function of GSN + Inflammatory Cancer‐Associated Fibroblasts in Renal Cell Carcinoma Immunotherapy: Insights From Pan‐Cancer Single‐Cell Landscape and Spatial Transcriptomics Analysis
Source: Cell Prolif. 2025 May 15;58(12):e70062. doi: 10.1111/cpr.70062 (PMC12686145; doi:10.1111/cpr.70062)

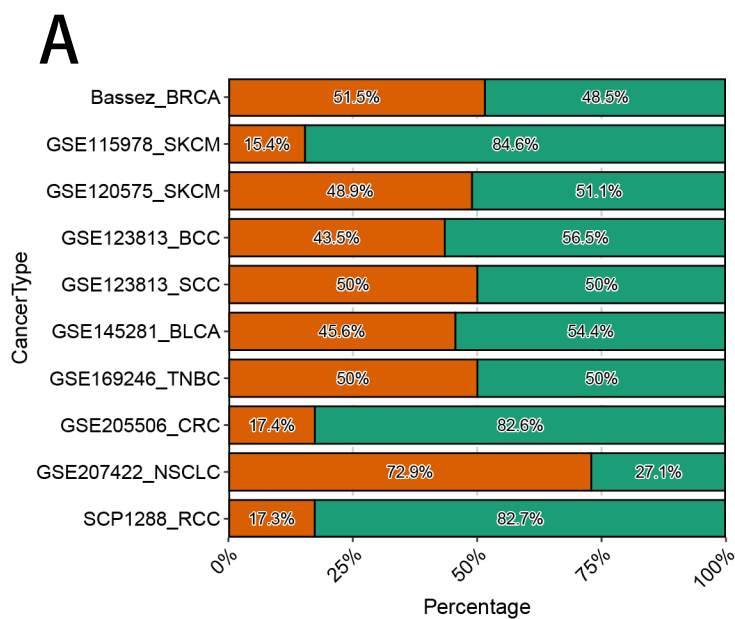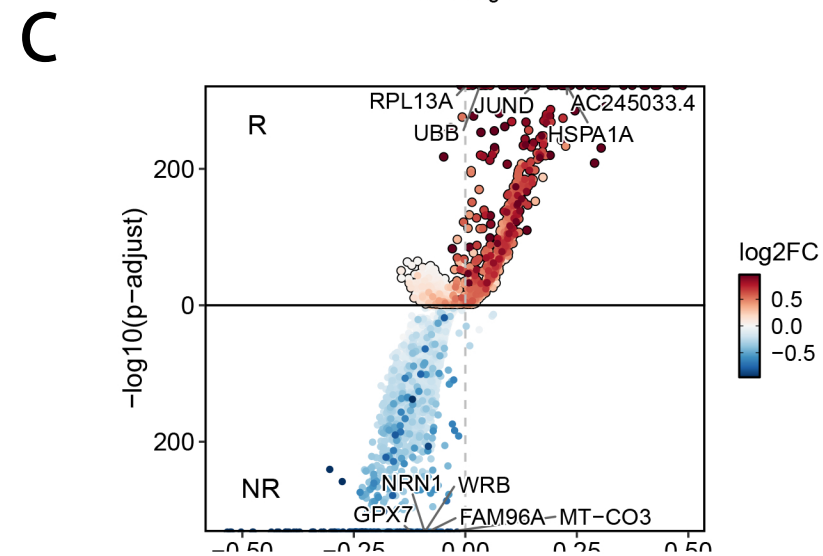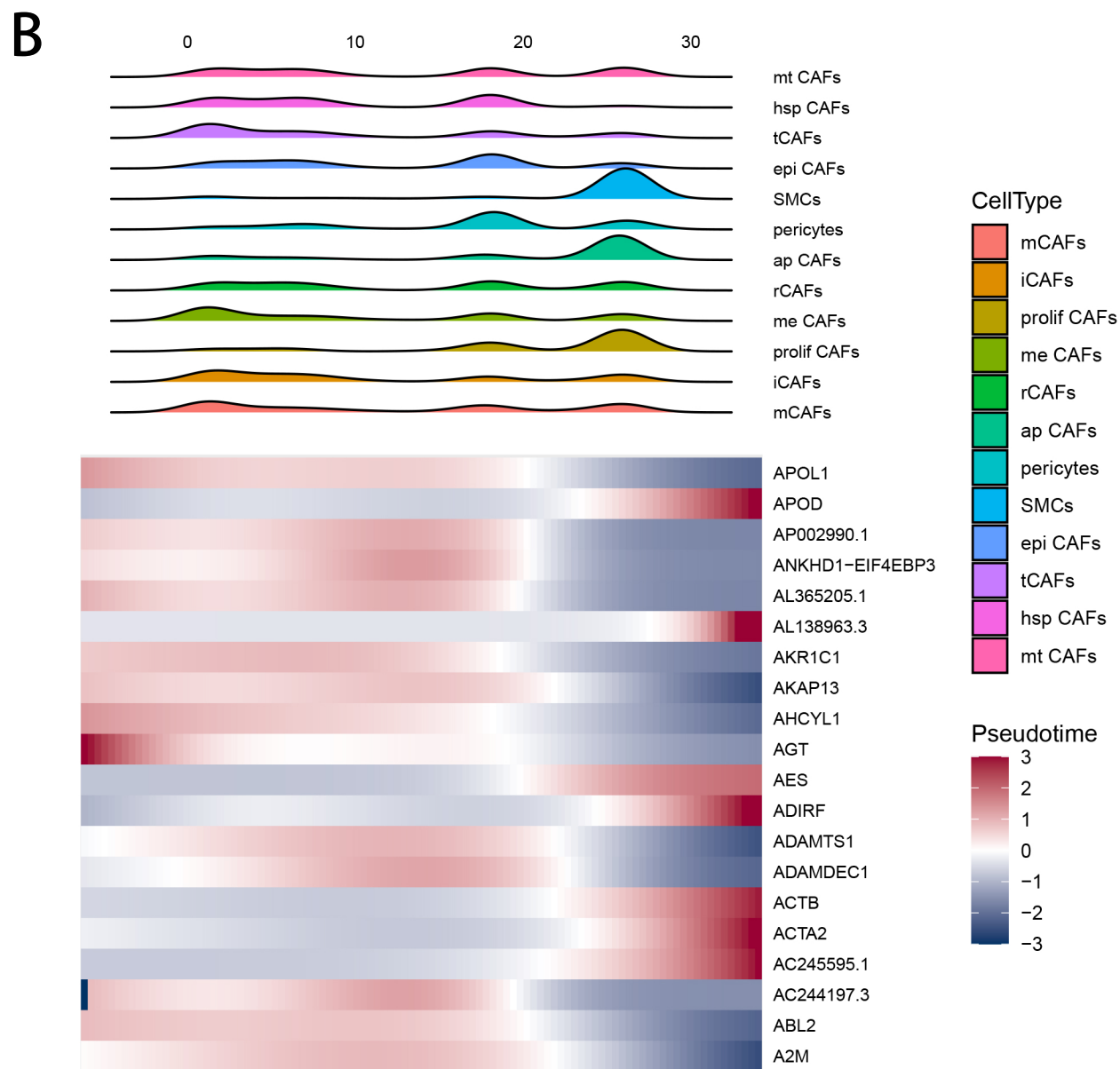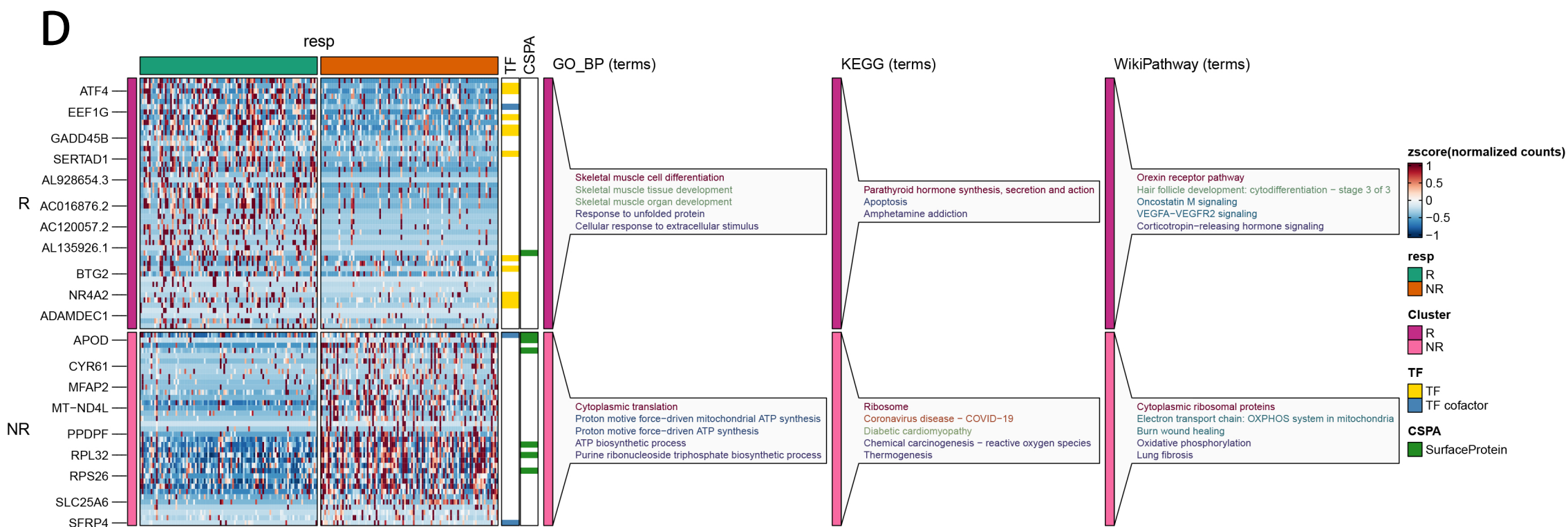

Supplement: Supplementary file 1 — Figure S1. (A) Different proportions of ICI treatment outcomes in nine scRNA‐seq cohorts. (B) Pseudotime DEGs identified by pseudotime analysis. (C) DEGs of fibroblasts in responders and non‐responders. (D) Distinction expression profiles of fibroblasts between responders and non‐responders. [file CPR-58-e70062-s008.pdf]

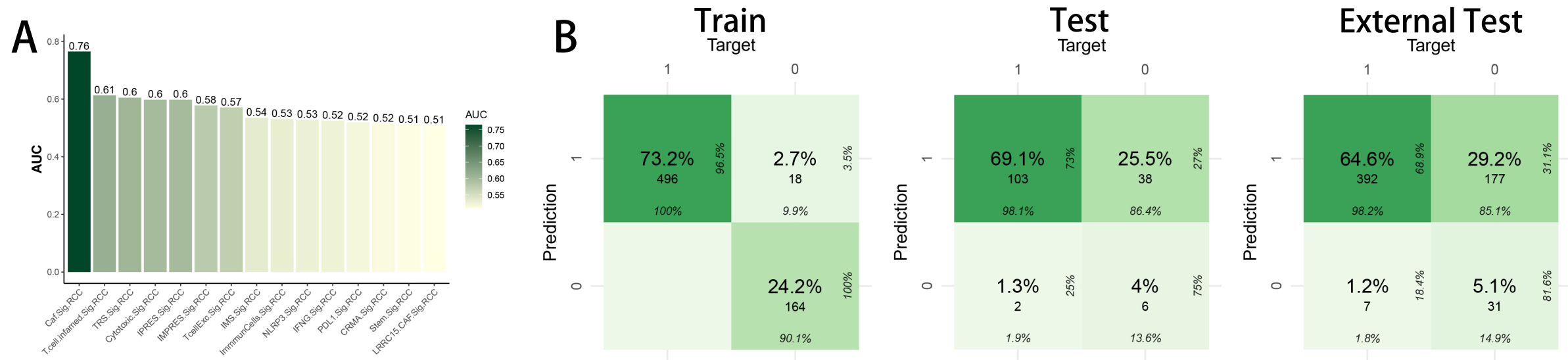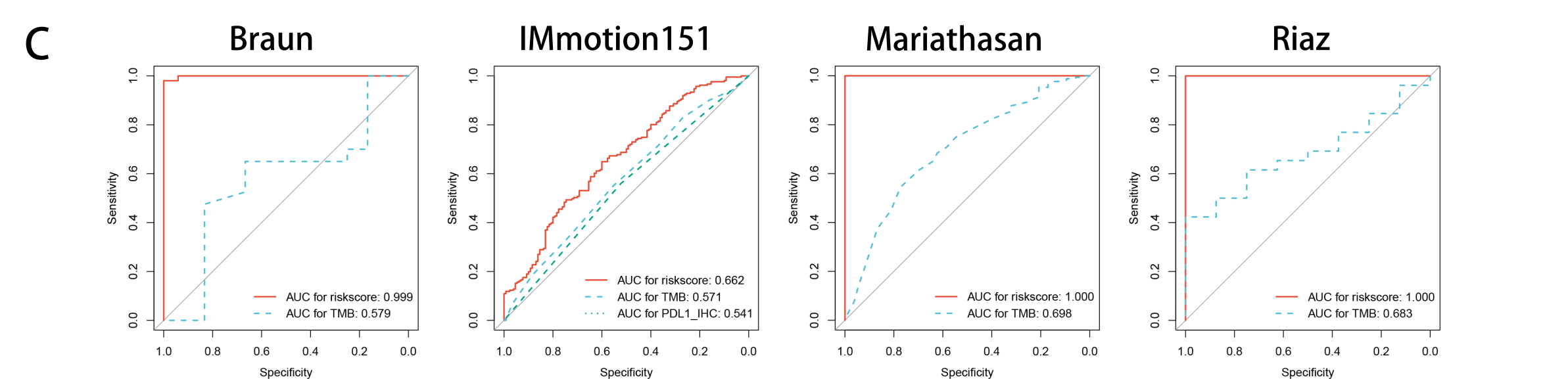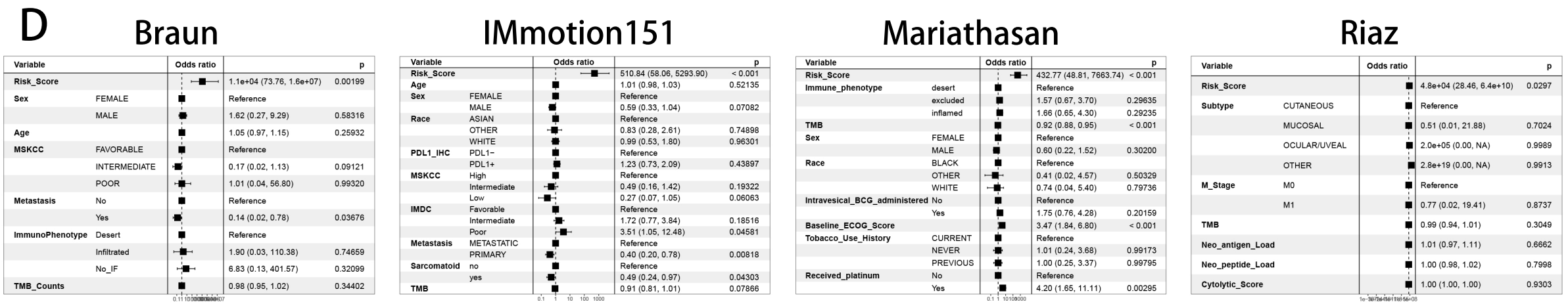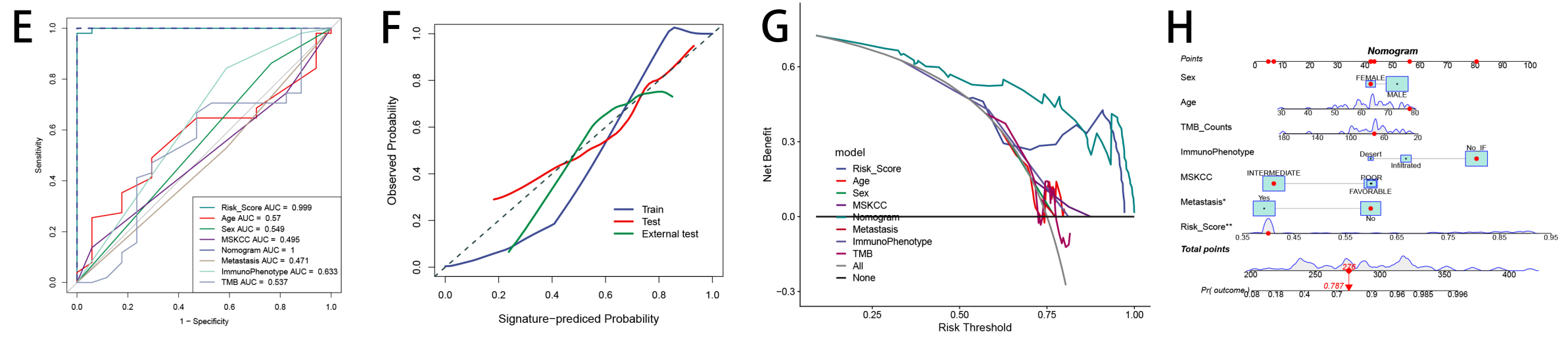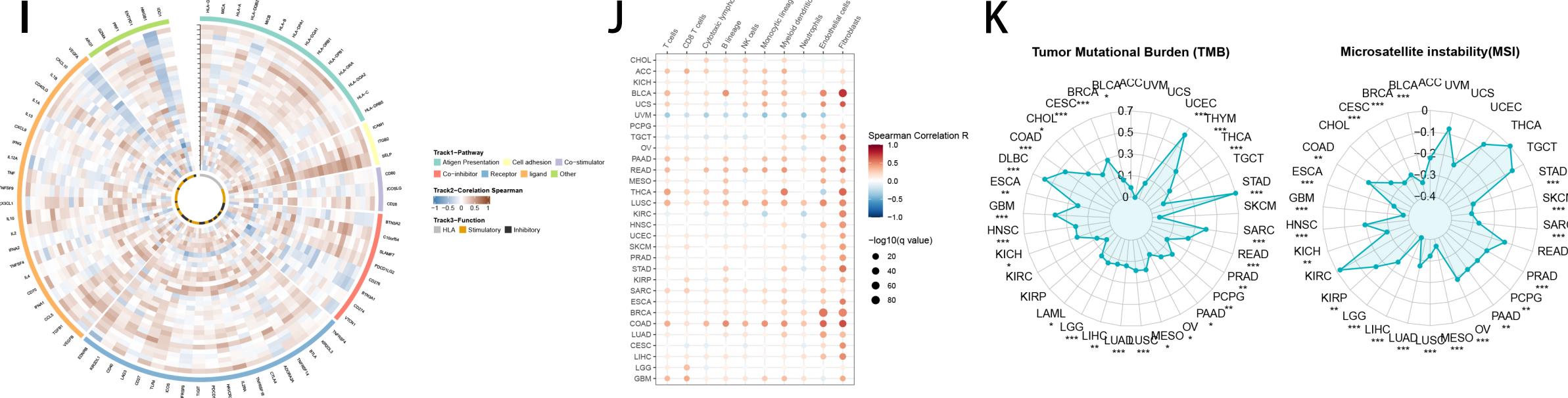

Supplement: Supplementary file 2 — Figure S2. (A) Comparison of the AUCs among the Caf.Sig model and other published signatures in IMmotion cohort. (B) Confusion matrix of the Caf.Sig Model in the training, internal validation and external validation cohorts. (C) Comparing Caf.Sig risk scores with TMB and PDL‐1 based on ROC Curves in IMmotion151, Mariathasan, Braun and Riaz cohorts. (D) Multivariate logistic regression analysis in IMmotion151, Mariathasan, Braun and Riaz cohorts. (E) AUC of the Caf.Sig Model, several clinical variables and the nomogram model in the Braun cohort. (F) Calibration curves in the training, internal validation and external validation cohorts. (G) DCA curves of the Caf.Sig Model, several clinical variables and the nomogram model in the Braun cohort. (H) The nomogram model in the Braun cohort. (I) Pan‐cancer analysis of associations among the Caf.Sig risk score and the expressions of immune‐related genes. (J) Pan‐cancer analysis of associations among the Caf.Sig risk score and various immune cell abundances. (K) Pan‐cancer analysis of associations among the Caf.Sig risk score and tumour mutational burden (TMB), and microsatellite instability (MSI) status. [file CPR-58-e70062-s004.pdf]

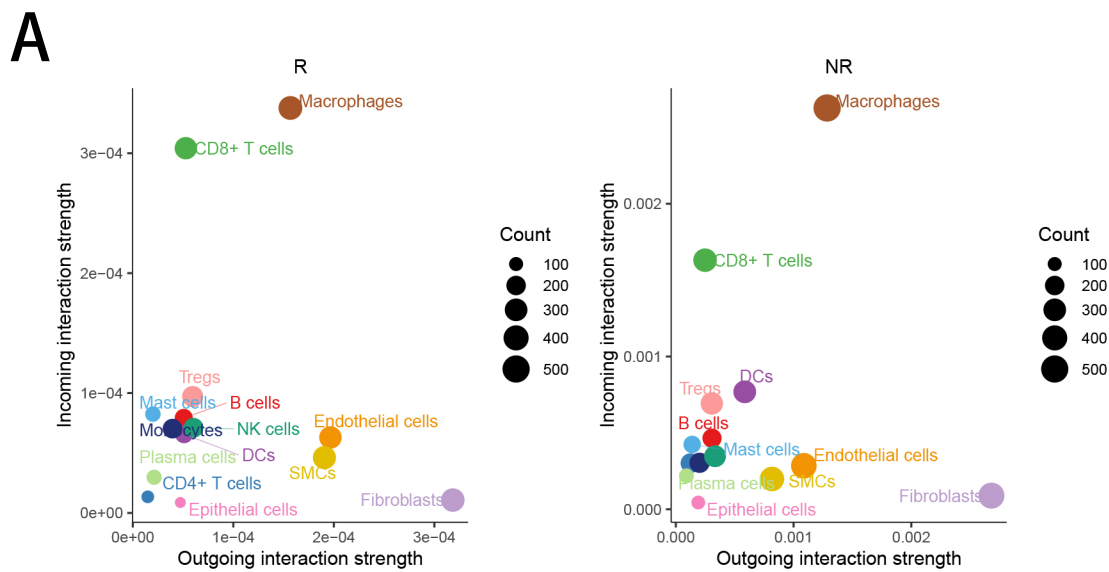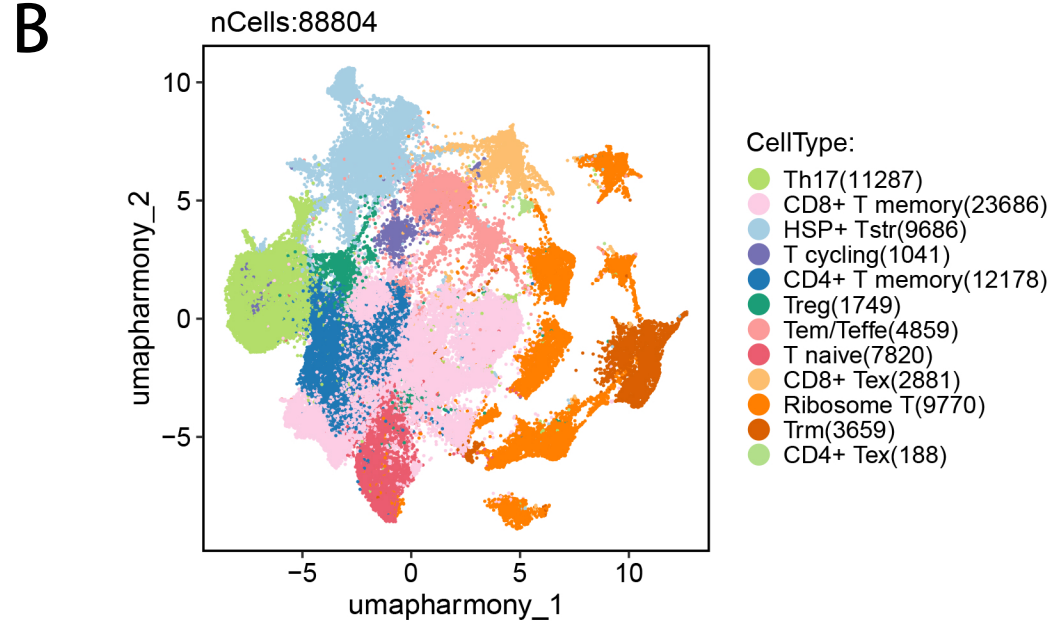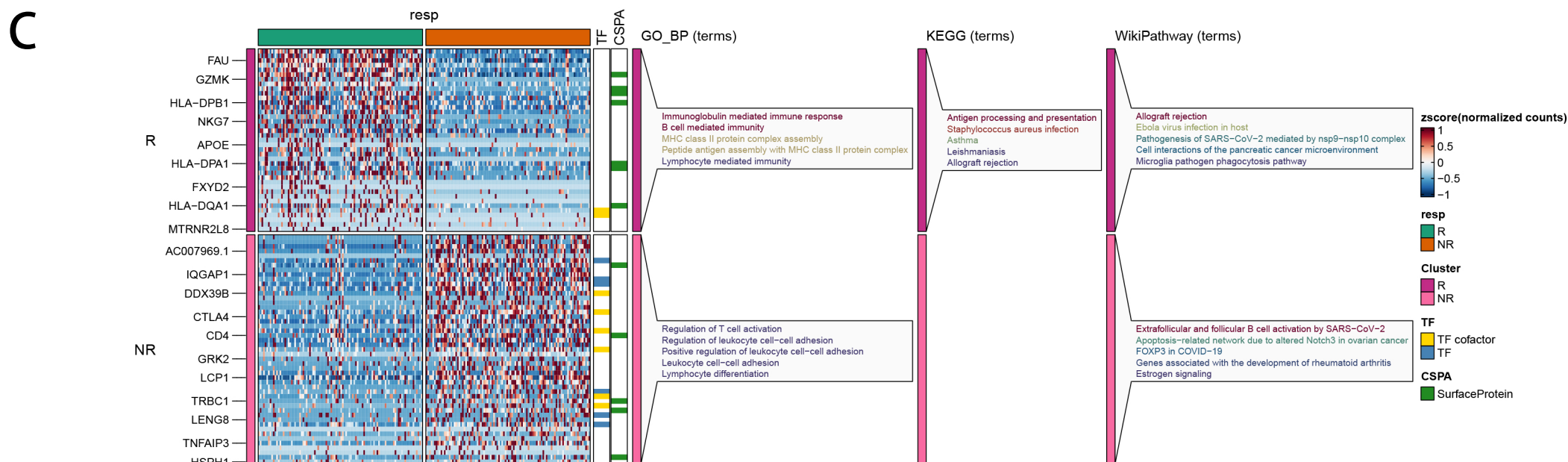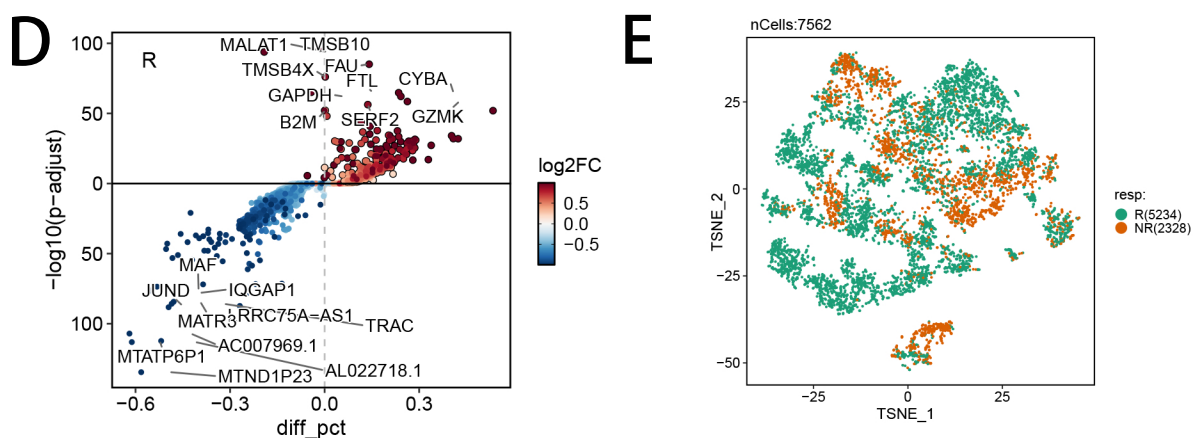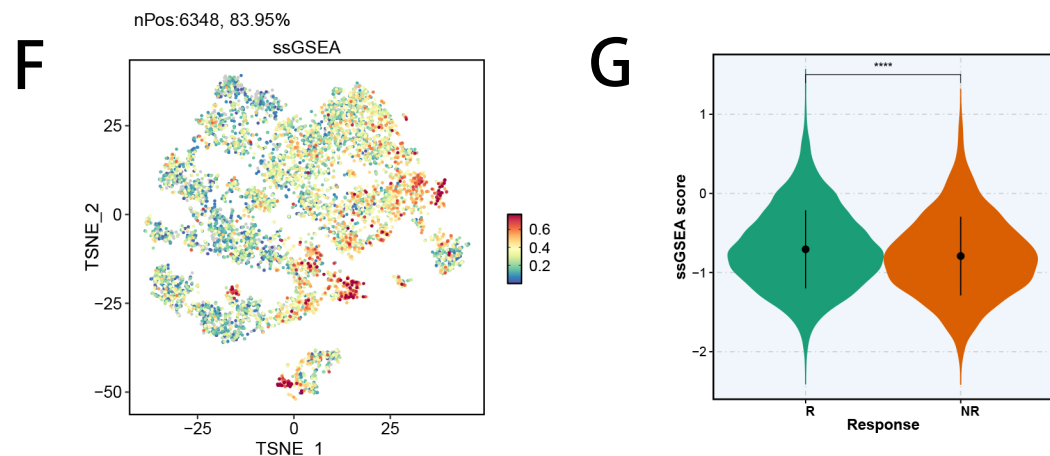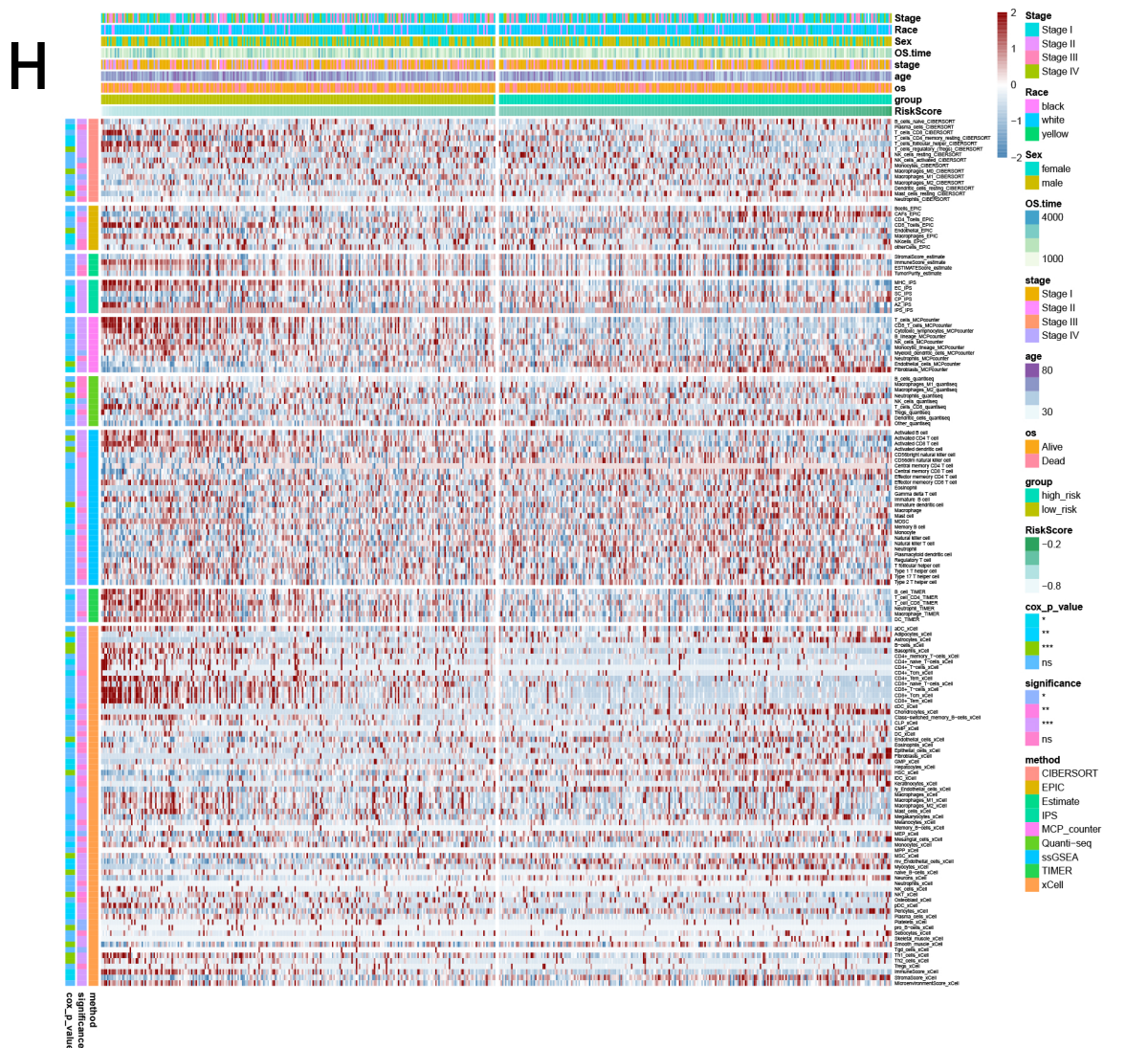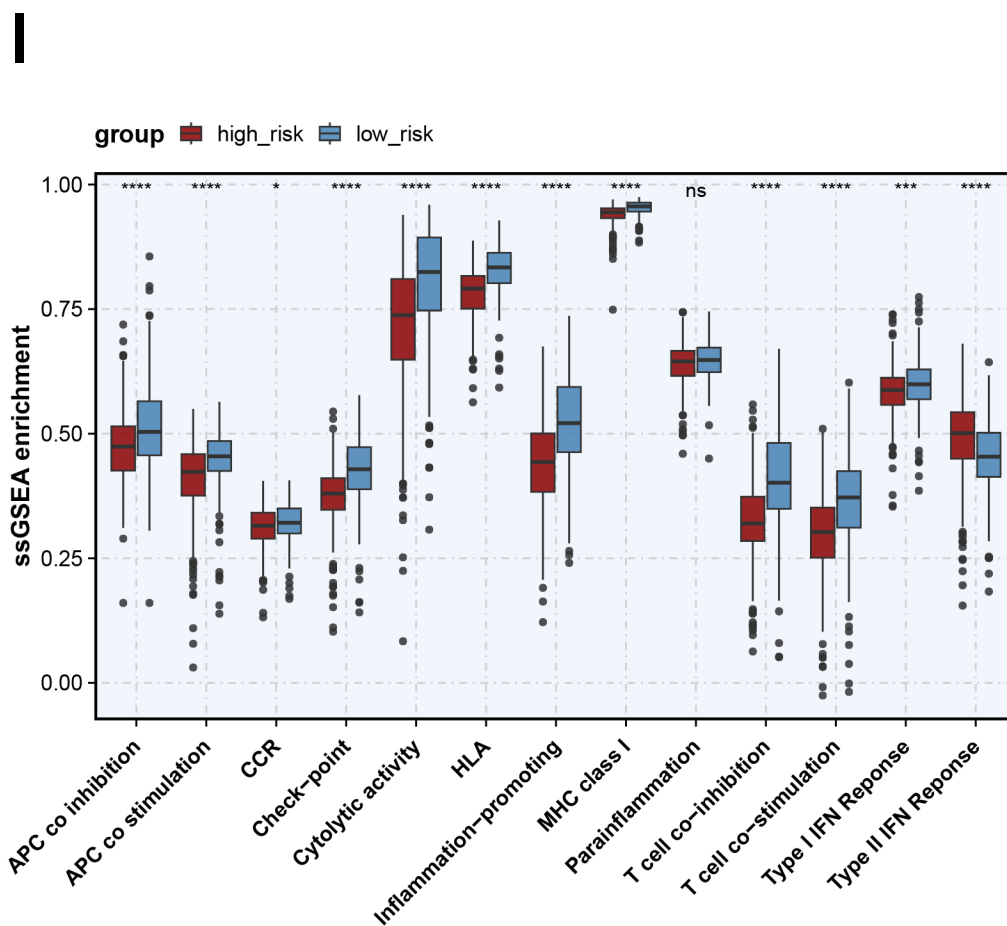

Supplement: Supplementary file 4 — Figure S4. (A) The relationship between differential outgoing interactions and incoming interaction strength for major cell types in responders and non‐responders. (B) Colour‐coded UMAP plot of T cell subgroups. (C) Distinction expression profiles of CD8+ Tex cells between responders and non‐responders. (D) DEGs of CD8+ Tex cells between responders and non‐responders. (E) Colour‐coded UMAP plot of responders and non‐responders in Tem/Teffe cells. (F) Effector score of Tem/Teffe cells in UMAP. (G) Comparison of effector score of Tem/Teffe cells between responders and non‐responders. (H) Differential immune cell abundances in patients with high or low iCAF scores in TCGA‐KIRC cohort. (I) Disparate immune function levels in patients with high or low iCAF scores in TCGA‐KIRC cohort. [file CPR-58-e70062-s001.pdf]

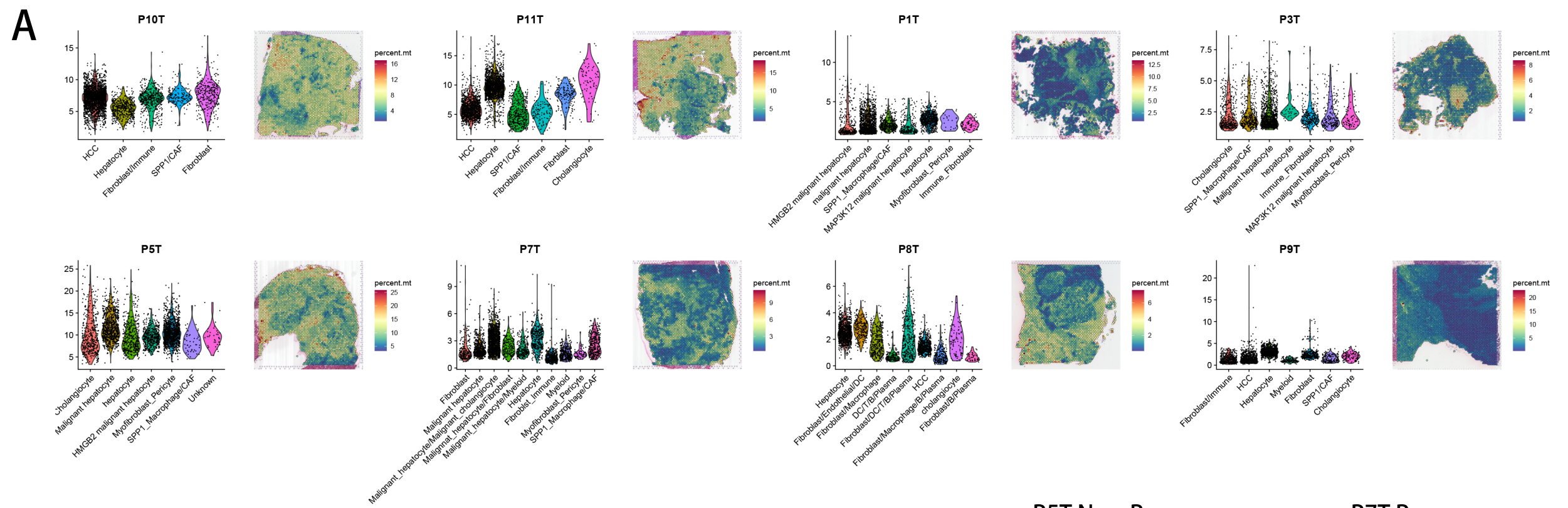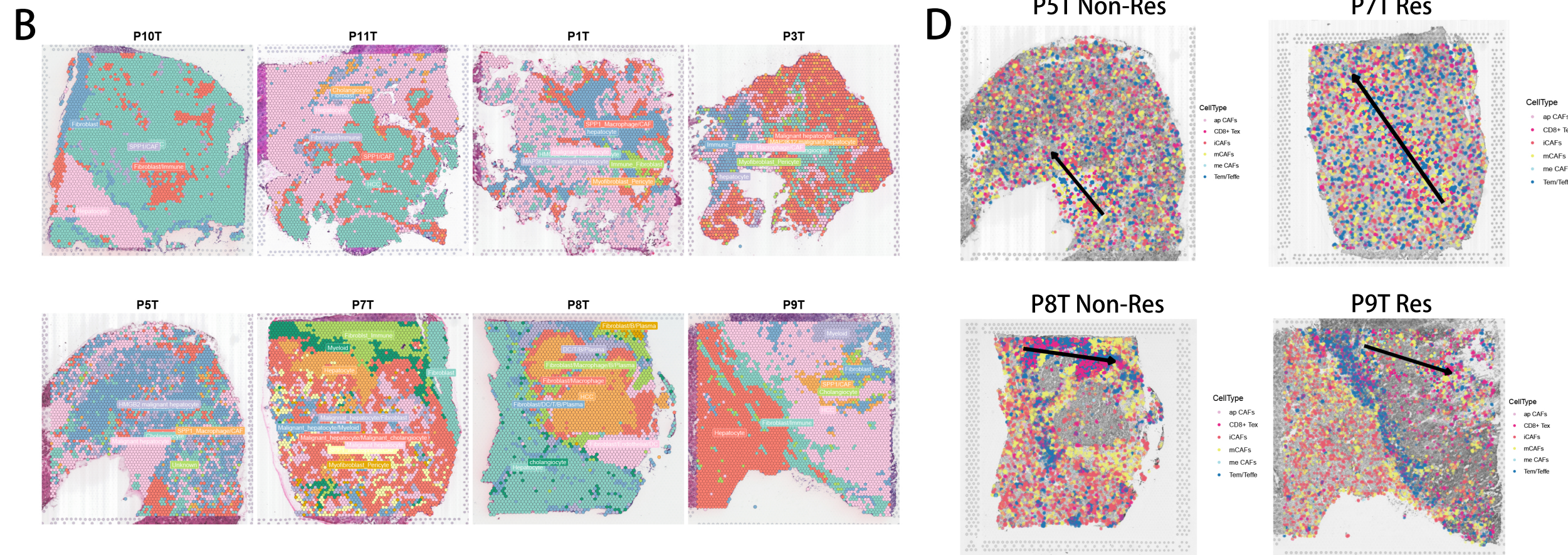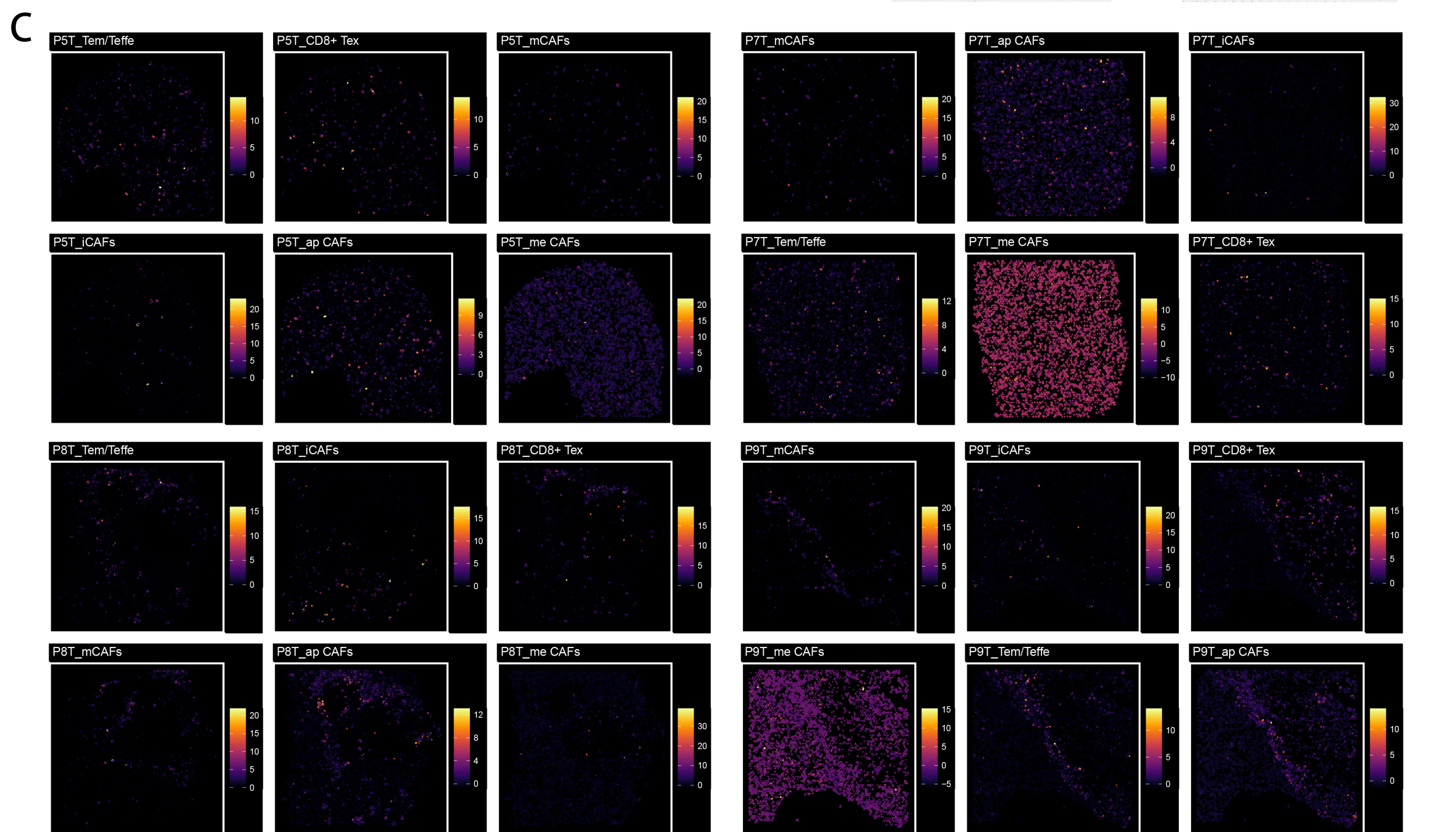

Supplement: Supplementary file 5 — Figure S5. (A) Violin plots show the quality control feature of percentage of mitochondrial genes in each patient. (B) Unbiased clustering of ST spots and cell types of each cluster in each patient. (C) High‐density regions of every cell subpopulation in each tumour section. (D) Spatial trajectory from high‐density areas of Tem/Teffe to high‐density areas of CD8+ Tex in four tumour sections. [file CPR-58-e70062-s007.pdf]

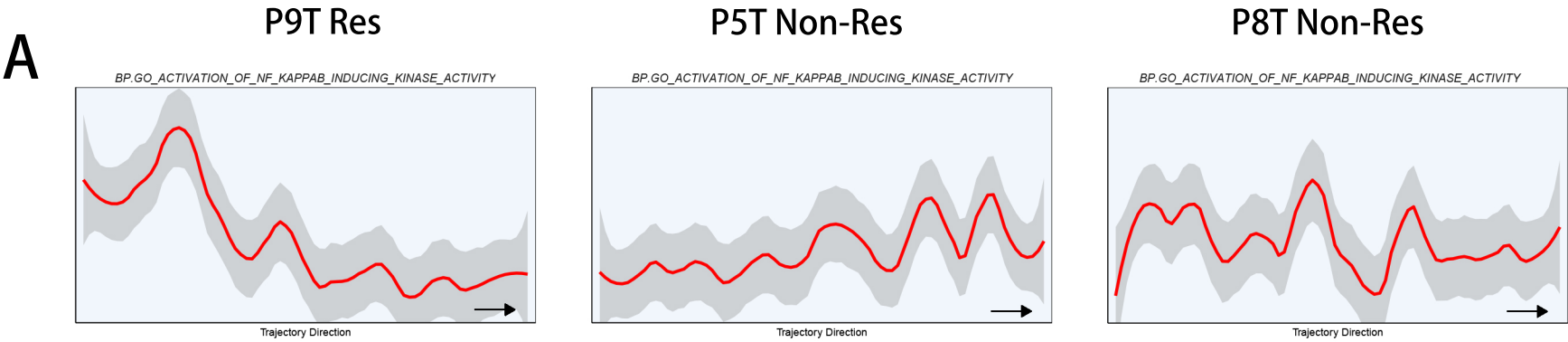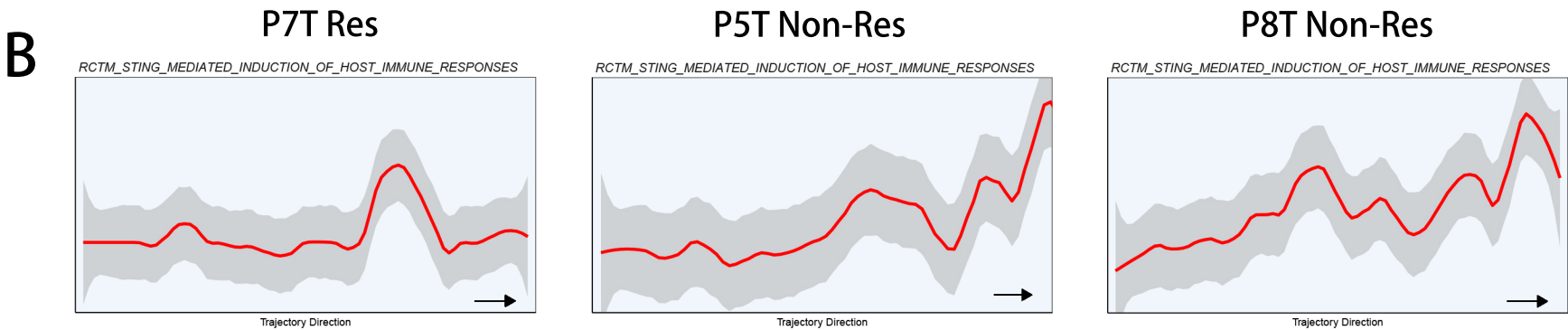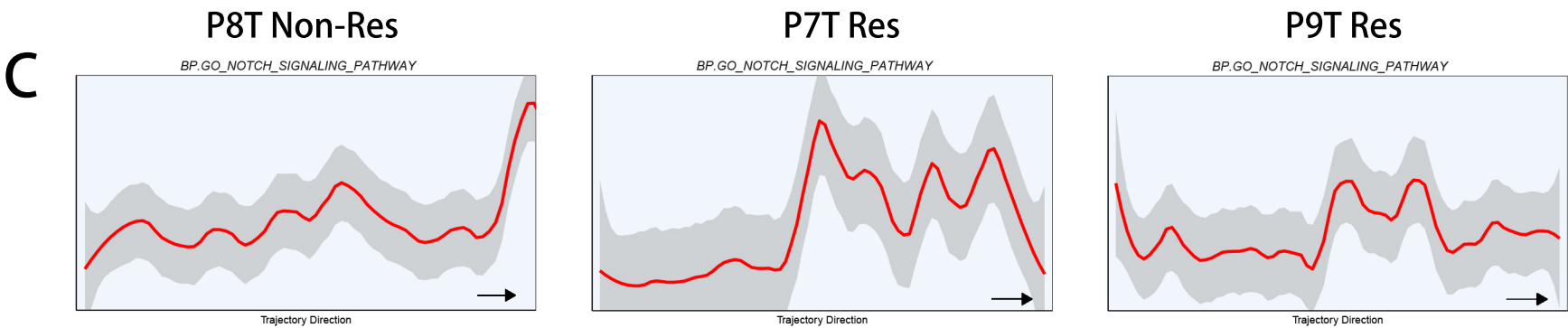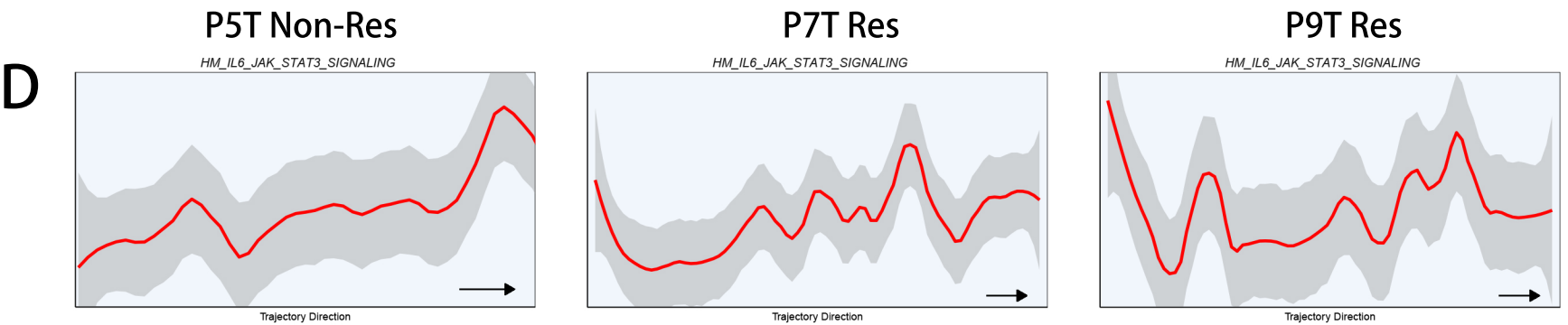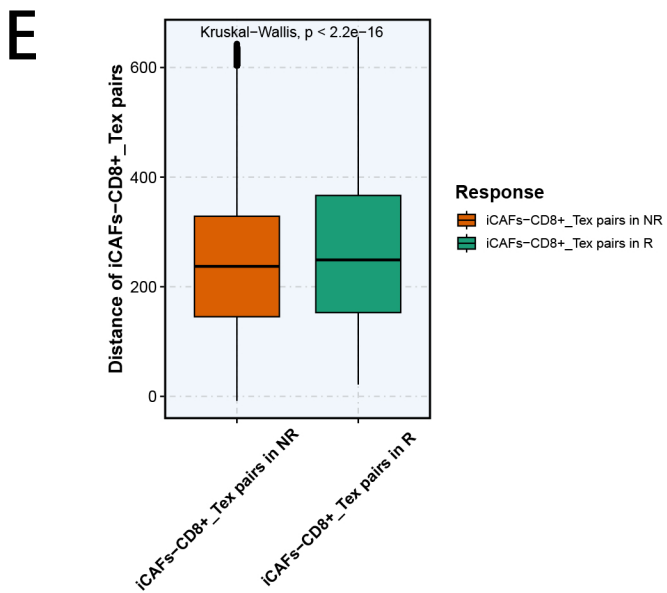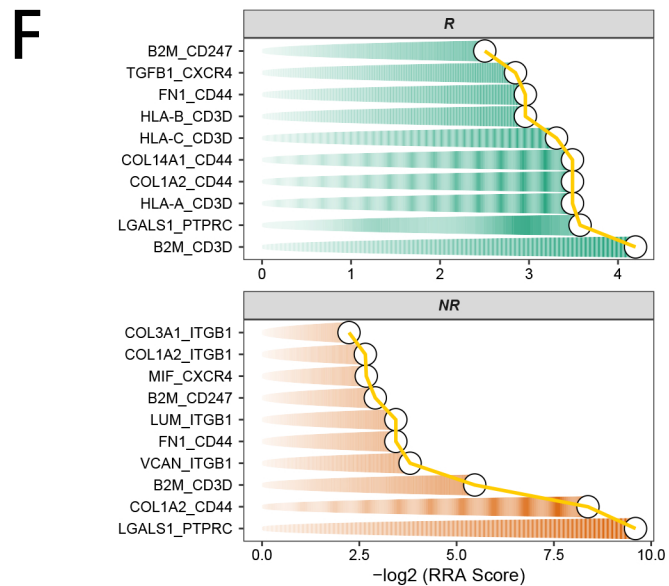

Supplement: Supplementary file 6 — Figure S6. (A) Changes in NF kappa B pathway activity of six cell subtypes along the trajectory direction of Tem/Teffe shifting to CD8+ Tex in three tumour sections. (B) Changes in STING pathway activity of six cell subtypes along the trajectory direction of Tem/Teffe shifting to CD8+ Tex in three tumour sections. (C) Changes in NOTCH pathway activity of six cell subtypes along the trajectory direction of Tem/Teffe shifting to CD8+ Tex in three tumour sections. (D) Changes in IL6_JAK_STAT3 pathway activity of six cell subtypes along the trajectory direction of Tem/Teffe shifting to CD8+ Tex in three tumour sections. (E) The interplay distances of LGALS1‐PTPRC in non‐responders and responders. (F) Integrated ranking of ligand‐receptor interactions based on interplay distances between iCAFs and CD8+ Tex using RRA algorithm in responders and non‐responders. The smaller the RRA score of a certain ligand‐receptor interaction, the closer it is between iCAFs and CD8+ Tex. [file CPR-58-e70062-s005.pdf]

A

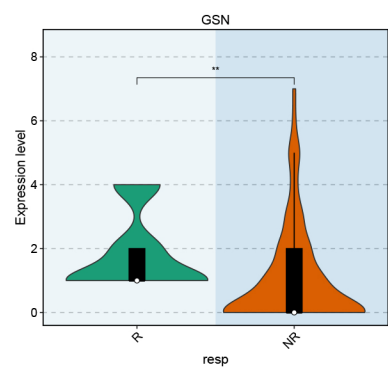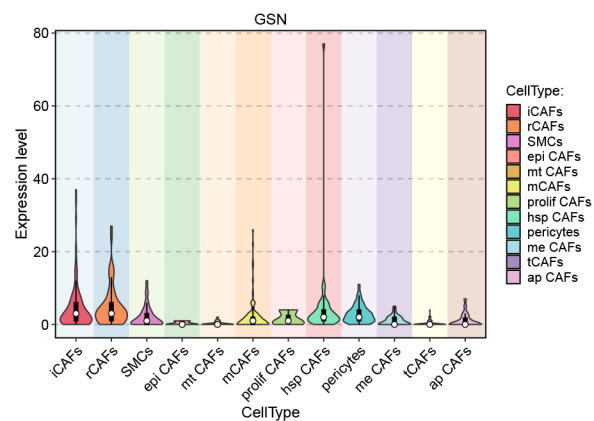

B

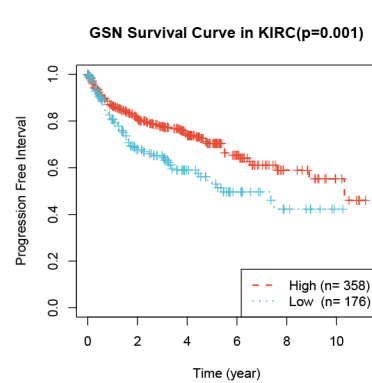

C

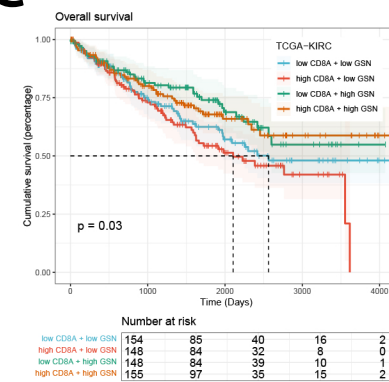

D

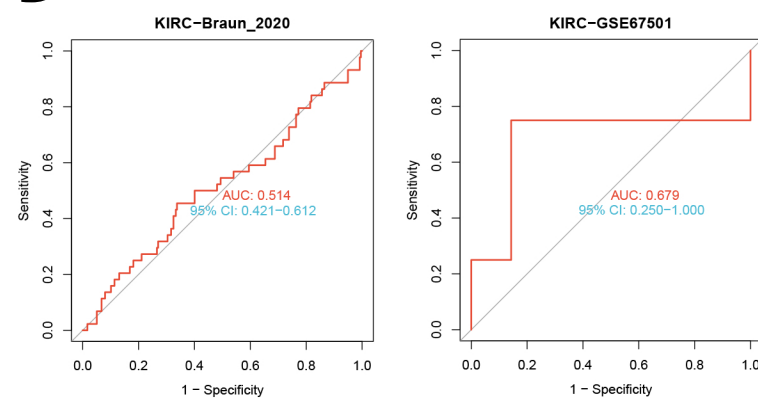

E

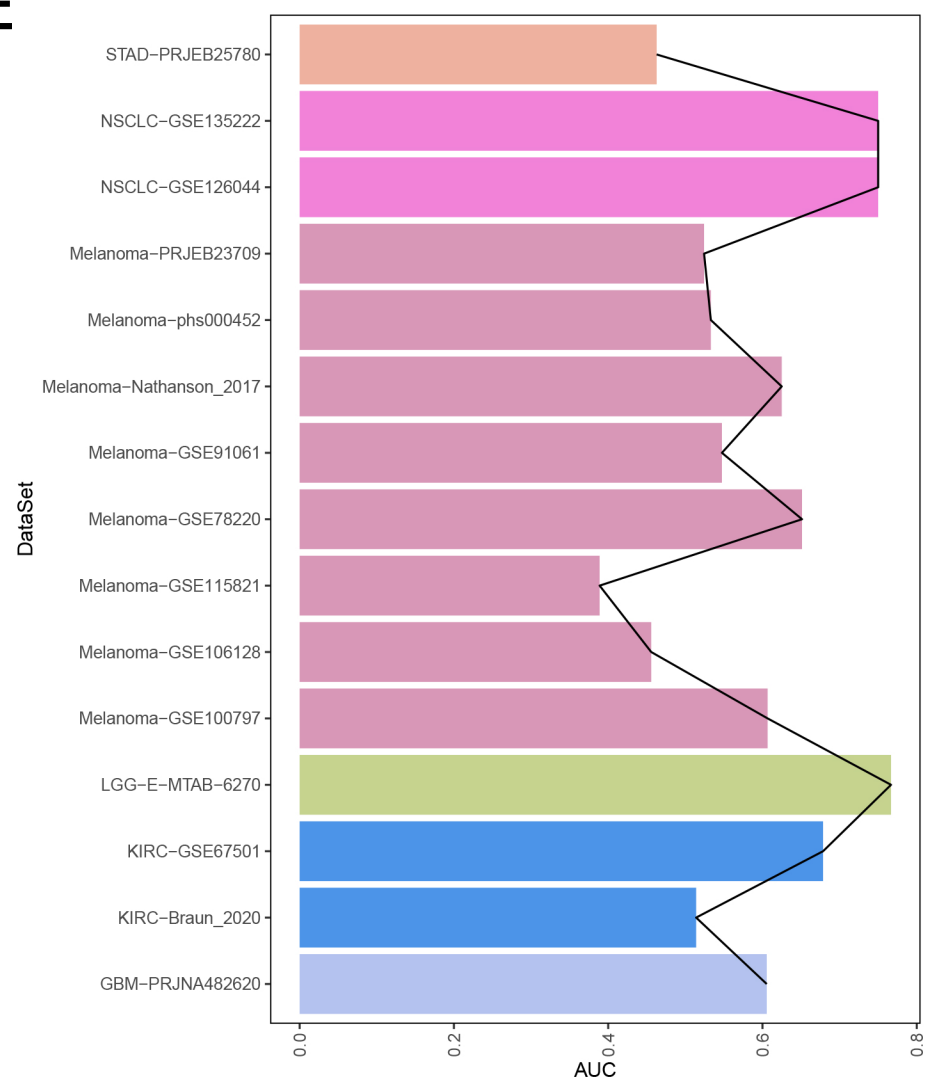

F

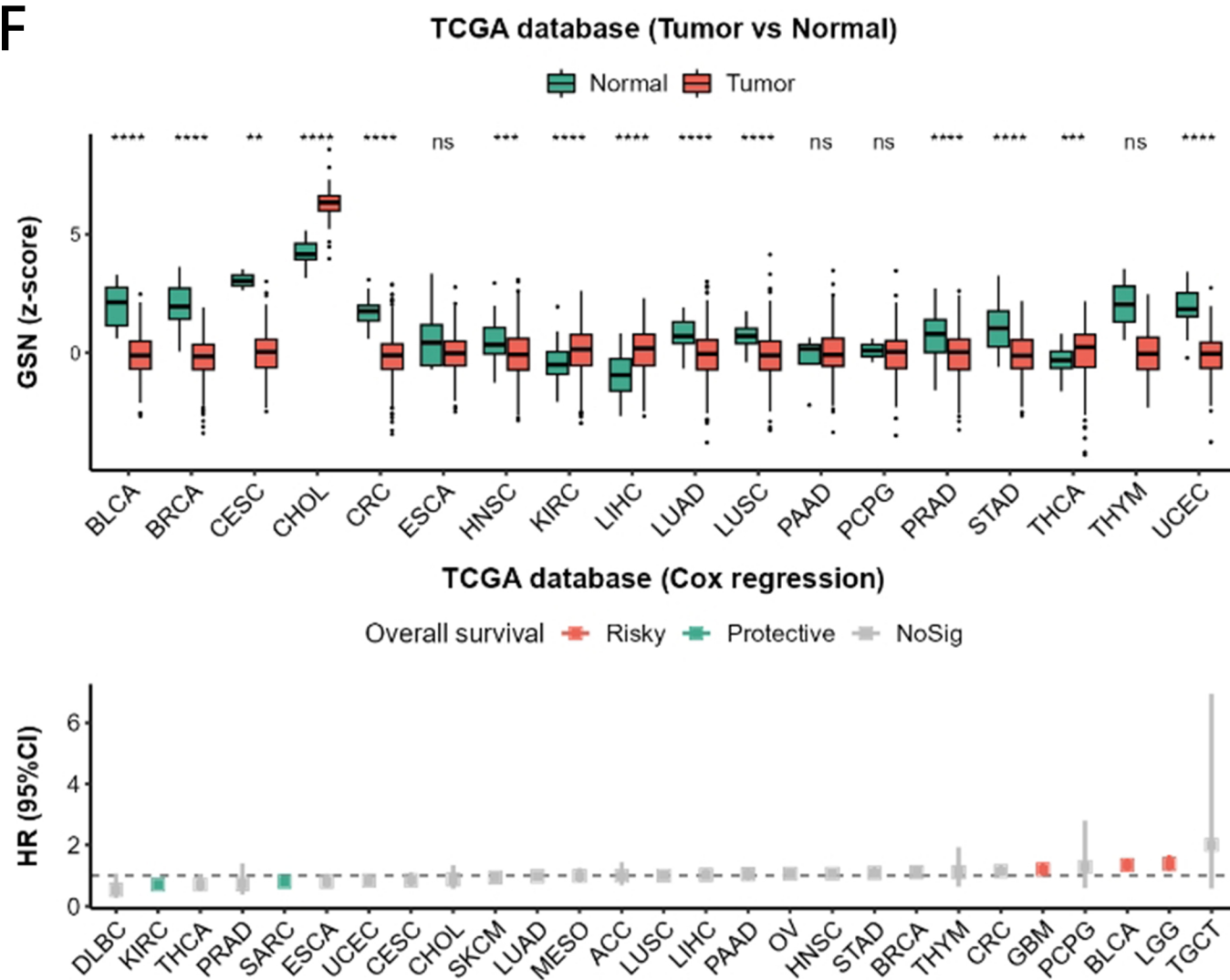

G

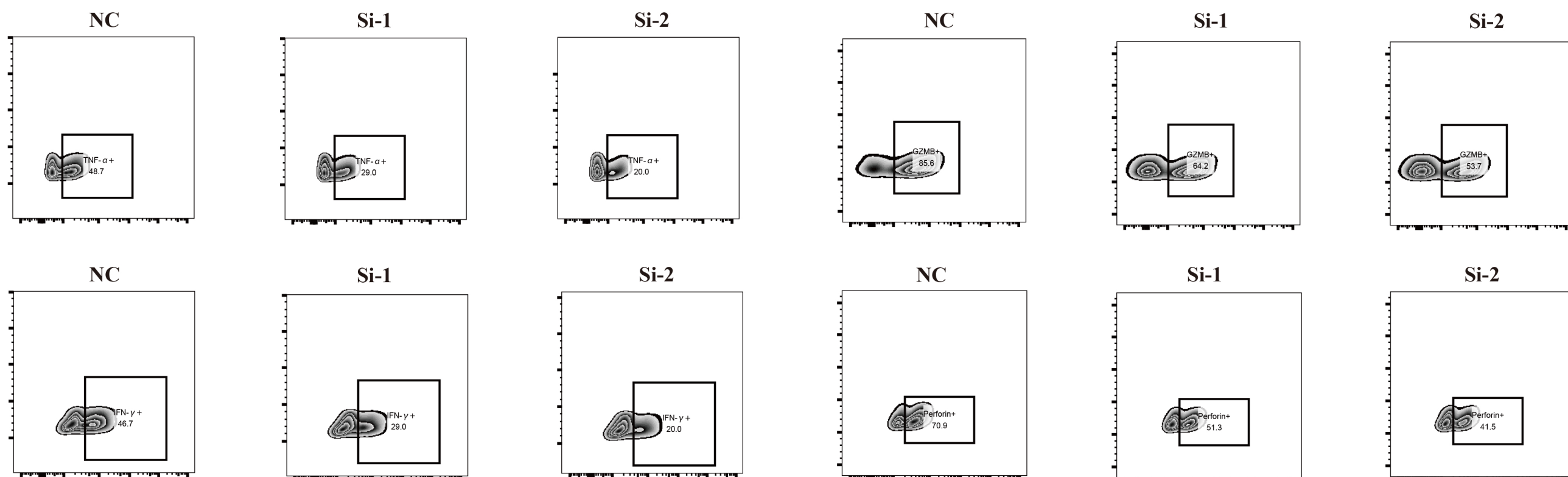

Supplement: Supplementary file 7 — Figure S7. (A) Expression of GSN in iCAFs from responders and non‐responders in RCC scRNA‐seq cohort, as well as GSN expression in every CAF subpopulation in RCC scRNA‐seq cohort. (B) Survival analysis of GSN (in terms of PFS) in TCGA‐KIRC cohort. (C) Survival analysis of GSN and CD8A (in terms of OSS) in TCGA‐KIRC cohort. (D) AUC value of GSN to predict ICI response in two RCC cohorts. (E) AUC value of GSN to predict ICI response in pan‐cancer RNA‐seq cohorts. (F) Pan‐cancer RNA‐seq analysis of GSN expression in tumoral and normal samples, as well as its prognosis value. (G) Representative flow cytometry figure of IFN‐γ, TNF‐α, GZMB and Perforin in CD8+ T cells isolated from the co‐culture system. [file CPR-58-e70062-s002.pdf]

- Ctrl
- AAV-GSN
- $\alpha$ PD-1
- AAV-GSN +  $\alpha$ PD-1

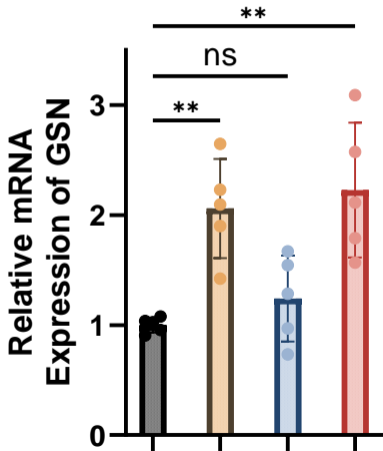

Supplement: Supplementary file 8 — Figure S8. qRT‐PCR analysis of GSN mRNA in tumours from four groups (Ctrl, AAV‐GSN, αPD‐1 and AAV‐GSN + αPD‐1). [file CPR-58-e70062-s006.pdf]
