# Supplementary material for: Deciphering the Immunomodulatory Function of GSN + Inflammatory Cancer‐Associated Fibroblasts in Renal Cell Carcinoma Immunotherapy: Insights From Pan‐Cancer Single‐Cell Landscape and Spatial Transcriptomics Analysis
Source: Cell Prolif. 2025 May 15;58(12):e70062. doi: 10.1111/cpr.70062 (PMC12686145; doi:10.1111/cpr.70062)
Supplement: Supplementary file 3 — Figure S3. (A) Visualising marker genes of each CAF subtype by dotplot. (B) Visualising marker genes of each CAF subtype by heatmap, as well as enrichment analysis results of each CAF subtype by GO and KEGG. (C) Expression of model genes from the Caf.Sig model in CAF subpopulations. (D) Utilising other six types of scRNA‐seq scoring algorithms (AUCell, Ucell, GSVA, singscore, AddModuleScore and PercentageFeatureSet) to validate the top Caf.Sig.Score of iCAFs. (E) Comparison of immune evasion and immune checkpoint genes among CAF subclusters. (F) Comparison of transcription factor activities among CAF subclusters. [file CPR-58-e70062-s003.pdf]

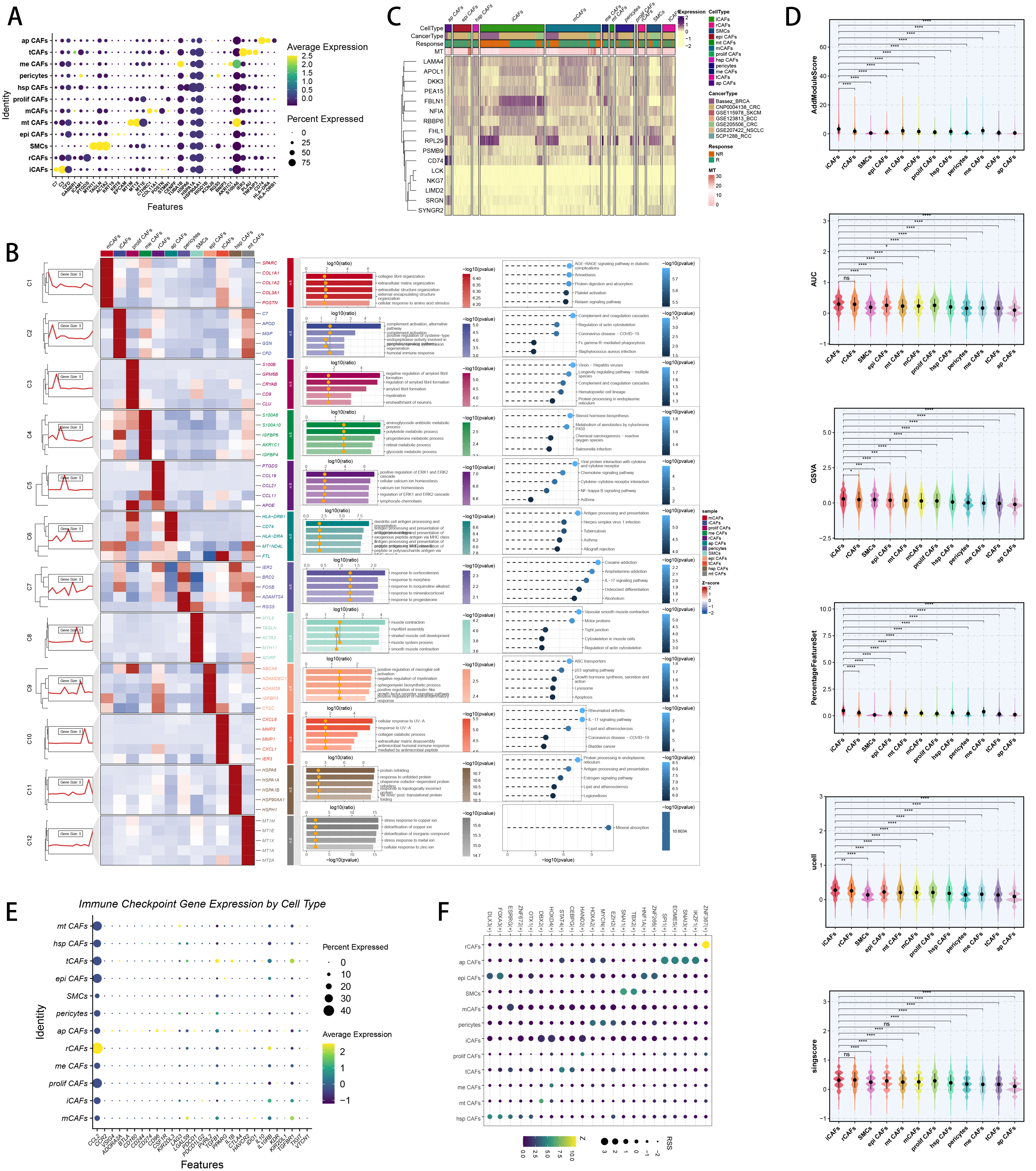

A
B
C
D
E
F
Average Expression
Percent Expressed
Identity
Features
ap CAFs
tCAFs
me CAFs
pericytes
hsp CAFs
prolif CAFs
mCAFs
mt CAFs
epi CAFs
SMCs
rCAFs
iCAFs
CellType
CancerType
Response
MT
Expression
LAMA4
APOL1
DKK3
PEA15
FBLN1
NFIA
RBBP6
FHL1
RPL29
PSMB9
CD74
LCK
NKG7
LIMD2
SRGN
SYNGR2
CancerType
Bassez_BRCA
CNP0004138_CRC
GSE115978_SKCM
GSE123813_BCC
GSE205506_CRC
GSE207422_NSCLC
SCP1288_RCC
Response
NR
R
AddModuleScore
AUC
GSVA
PercentageFeatureSet
ucell
singscore
log10(ratio)
-log10(pvalue)
sample
Z-score
Immune Checkpoint Gene Expression by Cell Type
RSS
